# Tracing NAD^+^ metabolism uncovers adaptive coordination between host and microbiome during colitis

**DOI:** 10.21203/rs.3.rs-8195970/v1

**Published:** 2025-12-04

**Authors:** Abrar I. Alsaadi, Lina Welz, Anant A. Pothakamury, Clara Gilloteau, Praveena Prasad, Mahmoud S. Yahia, Jaedon Sadler, Jaclyn D. Smith, Brenita C. Jenkins, Garret S. Diven, Johanna Bornhäuser, Taous Mekdoud, Shuchang Tian, Philip Rosenstiel, Stefan Schreiber, Jordan E. Bisanz, Konrad Aden, Melanie R. McReynolds

**Affiliations:** 1 Department of Biochemistry and Molecular Biology, The Huck Institute of Life Sciences, Pennsylvania State University, University Park, Pennsylvania, USA; 2 Institute of Clinical Molecular Biology, Christian-Albrechts-University and University Hospital Schleswig-Holstein, Campus Kiel, Kiel, Germany; 3 Department of Internal Medicine l., Christian-Albrechts-University and University Hospital Schleswig-Holstein, Campus Kiel, Kiel, Germany

**Keywords:** NAD^+^, IBD, gut microbiota, tryptophan, nicotinamide, DSS colitis

## Abstract

Host-microbiota metabolic interactions critically regulate nicotinamide adenine dinucleotide (NAD^+^) homeostasis, and their disruption is increasingly linked to chronic diseases including inflammatory bowel disease (IBD). However, it remains unclear whether NAD^+^ dysregulation in IBD arises from impaired production, enhanced consumption, or both. Using multi-omics approaches and stable isotope-labeled NAD^+^ precursors administered via intravenous infusion in a murine model of dextran sulfate sodium (DSS)-induced colitis, we mapped tissue- and lumen-specific NAD^+^ metabolism under inflammatory stress. Our results reveal tissue-specific rewiring of NAD^+^ metabolism, with increased flux through the salvage pathway compensating for reduced *de novo* NAD^+^ synthesis from tryptophan. In parallel, microbial *de novo* NAD^+^ production was elevated, highlighting a cooperative host–microbiota response to inflammatory stress. These findings demonstrate differential regulation of NAD^+^ biosynthesis during acute colitis and underscore the dynamic interplay between host and microbial metabolism in maintaining NAD^+^ homeostasis under inflammatory conditions.

Interactions between the host and gut microbiota are critical for maintaining nicotinamide adenine dinucleotide (NAD^+^) homeostasis (1). NAD^+^ is a central coenzyme that regulates cellular metabolism and signaling, supporting processes such as energy production, DNA repair, and stress responses (2–4). In mammals, NAD^+^ can be synthesized *de novo* from tryptophan (Trp) through the kynurenine pathway (KP), Preiss-handler pathway from nicotinic acid (NA), from nicotinamide riboside (NR), but most of NAD^+^ synthesis is derived from nicotinamide (NAM) through the salvage pathway (5, 6). Despite its importance, the regulation and utilization of these pathways under pathological conditions remain poorly understood.

Recent studies highlight the gut microbiota’s role in supporting host NAD^+^ homeostasis through the bidirectional cycling of NAD^+^ precursors between the host tissues and gut microbiota under normal physiological conditions (1, 7). The gut microbiota contributes to host NAD^+^ synthesis by converting host-derived NAM to NA via bacterial nicotinamidase encoded by *PncA* gene, a process not found in mammals. Thus, the presence of NA in the GI tract is microbiome-dependent, which provides an alternative route for NAD^+^ synthesis in the host tissues and gut microbiota (1, 7). However, physiological stressors, such as infection or inflammation, can disrupt this bidirectional metabolic communication, leading to dysregulation of NAD^+^ biosynthesis and degradation pathways and potentially contributing to disease pathology.

Emerging evidence has linked altered NAD^+^ metabolism to intestinal inflammation, suggesting its dysregulation may contribute to the pathogenesis of inflammatory bowel disease (IBD) by altering immune function and tissue repair (8–12). IBD, including Crohn’s disease (CD) and ulcerative colitis (UC), is a chronic inflammatory condition of the gastrointestinal (GI) tract, leading to tissue damage, gut dysbiosis, and an altered gut environment (13). While significant progress has been made in understanding the immune and genetic mechanisms of IBD, the metabolic reprogramming that accompanies disease manifestation and progression remains poorly understood, despite its potential to drive disease pathology (14–16). Therefore, we hypothesized that acute colitis disrupts NAD^+^ homeostasis in both the host tissues and gut microbiota, resulting in increased NAD^+^ turnover driven by heightened inflammation-induced energy demand.

While the role of NAD^+^ in inflammation is recognized, there is limited understanding of how acute inflammation, such as that seen in DSS-induced colitis, alters NAD^+^ biosynthesis and its pathways in a tissue-specific manner. In this study, we leverage a multi-omics approach using isotope tracing via intravenous infusions, metabolomics, and metagenomics– to explore how the host and gut microbiome coordinate NAD^+^ biosynthesis under normal physiological and inflammatory conditions. Using stable isotope tracers of tryptophan and nicotinamide, we aimed to determine how acute colitis reprograms NAD^+^ metabolism across different tissues and within the gut microbiome, with particular emphasis on the activation of *de novo* and salvage biosynthetic pathways.

By integrating complementary omics approaches, we identified dynamic metabolic changes that disrupt NAD^+^ homeostasis and contribute to disease progression. Our analysis demonstrates that acute DSS-induced colitis rewires NAD^+^ metabolism, activating nicotinamide (NAM)–driven salvage pathways in a tissue-specific manner as a compensatory response to sustain NAD^+^ levels during intestinal inflammation. This previously unrecognized connection between dysregulated NAD^+^ metabolism and intestinal inflammation highlight potential therapeutic avenues to restore metabolic balance, improve intestinal health, and enhance patient outcomes.

## Results:

### Inflammation and Tissue Damage Associates with Disrupted Host and Microbial NAD^+^ Metabolism in Acute Colitis

**Fig.1|:**
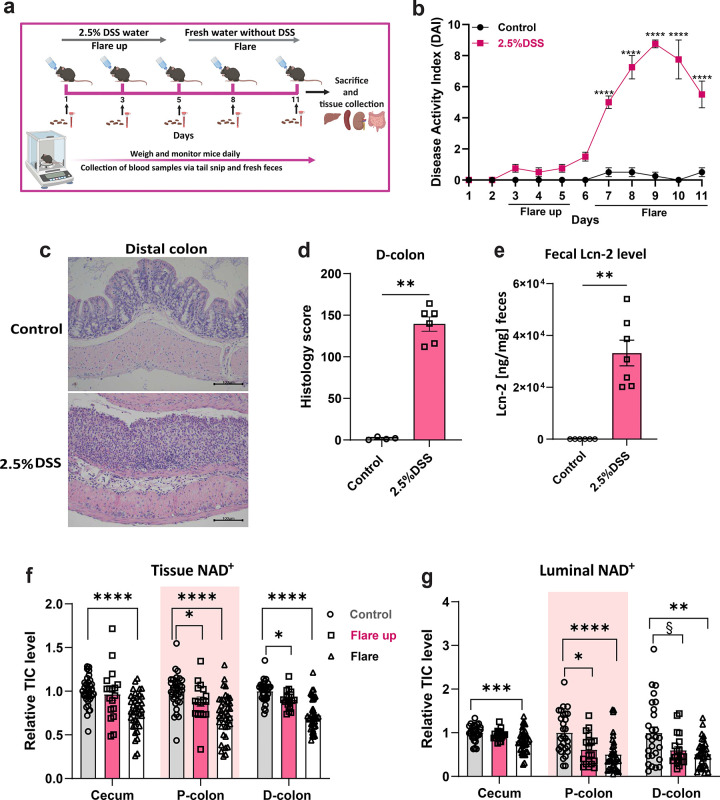
Acute-induced colitis decreases NAD^+^ levels in colonic tissues. **(a)** Experimental setup illustrating the administration of 2.5% DSS dissolved in water for 5 days, followed by a switch to normal drinking water until the end of the experiment (day 11) in 11–12 weeks old C57BL/6 male mice. Longitudinal sampling of fecal and blood samples (shown in black arrows) was collected for LC-MS metabolomics and metagenomics analysis. At the end of the experiment mice were sacrificed by cervical dislocation, tissues and luminal samples were dissected for LC-MS metabolomics. **(b)** Daily recorded disease activity index (DAI) in DSS-treated and control mice including weight loss compared to the initial weight, stool consistency, bloody stool, rectal bleeding, overall activity, posture, and fur. **(c)** Representative image of the H&E staining of Swiss roll sections of the distal colon segment (at magnification 20x, scale bar 100μm), and **(d)** a corresponding histopathological score. **(e)** Fecal lcn-2 was measured by ELISA in fecal samples collected on days 8 and 11 during the flare phase of intestinal inflammation and controls. LC-MS relative total ion count (TIC) levels of NAD^+^ pooled from all experiments in this study of (**f**) tissues NAD^+^ levels and (**g**) luminal NAD^+^ levels. Data are presented as mean ± SEM; in (**f**) (n= 18–40) and in (**g**) (n=20–40). Statistical significance was determined by Kruskal-Wallis test with post hoc Dunn’s test for comparisons among more than two groups and Mann-Whitney U test for two-groups comparisons. § <0.1, *P<0.05, **P<0.01, ***P<0.001, and ****P<0.0001. (a) was created with Biorender.com

Acute colitis was induced in mice by administrating 2.5% dextran sulfate sodium (DSS) in drinking water for 5 days, followed by normal water until day 11 (Figure 1a) (17, 18). This approach allowed us to monitor disease activity and identify how flux of NAD^+^ is impacted during the flare up (early) and flare (active) phases of acute induced colitis. The early flare up phase is characterized by mild colitis occurring on days 4–5, and the active flare phase spans from days 7 to 11, in which the colon is fully inflamed (19–21) (Figure S1a). DSS treated mice showed a substantial weight loss (Figure S1b), and increased disease activity index (DAI) during the active flare phase compared to the early flare up phase and control mice (18, 22) (Figure 1b). The induction of acute colitis resulted in structural alterations of the epithelial and mucosal layer in the distal colon (D-colon) of DSS-treated mice, leading to a higher score for histological inflammation compared to control mice (Figure 1c–d). To further confirm the induction of colitis, we measured the levels of fecal lipocalin-2 (Lcn-2), also known as a neutrophil gelatinase-associated lipocalin (NGAL), a biomarker of intestinal inflammation (23, 24). Fecal Lcn-2 levels were significantly elevated in mice treated with 2.5% DSS compared to control mice, reflecting increased intestinal inflammation, which was further supported by the upregulation of Lcn-2 expression in the D-colon (Figure 1e and S1c). We also observed a significant upregulation of pro-inflammatory cytokines, including *Ifn-γ*, *Tnf-α*, *Il-6*, *Il-1β* while *Il-1α* showed an upward trend (Figure S1d–h). We noted elevated levels of anti-inflammatory cytokines *Il-10* and *Il-22* in DSS-treated mice compared to controls in the D-colon (Figure S1i–j). Supporting previous observation, the total NAD^+^ pool was reduced in the cecum and colon during intestinal inflammation (Figure 1f). Additionally, DSS treatment in the active flare phase resulted in disrupted luminal NAD^+^ levels in the cecum and colon, indicating disrupted microbial NAD^+^ metabolism (Figure 1g).

### Increased NAD^+^ Synthesis from Tryptophan in Acute Induced Colitis

Tryptophan (Trp) is an essential amino acid metabolized via the kynurenine pathway (KP), leading to the production of quinolinic acid (QUIN) and subsequent NAD^+^ synthesis (Figure S2a). The KP is primarily mediated by hepatic tryptophan 2,3-dioxygenase (TDO), with a lesser contribution from extrahepatic indoleamine 2,3-dioxygenase (IDO) (25, 26). Previous studies have indicated that IBD is associated with alterations in Trp metabolism, which result in the increased conversion of Trp to Kyn due to elevated IDO enzyme activity and is reflected by an increased Kyn/Trp ratio (11, 12, 27, 28). Supporting this, we detected an increase of the circulating Kyn/Trp ratio during both the early flare up and active flare phases compared to control (29) (Figure S2b). We hypothesized that the increased Trp flux through the KP represents a compensatory response to inflammation, in which NAD^+^ demand is heightened during the immune response.

**Fig.2|:**
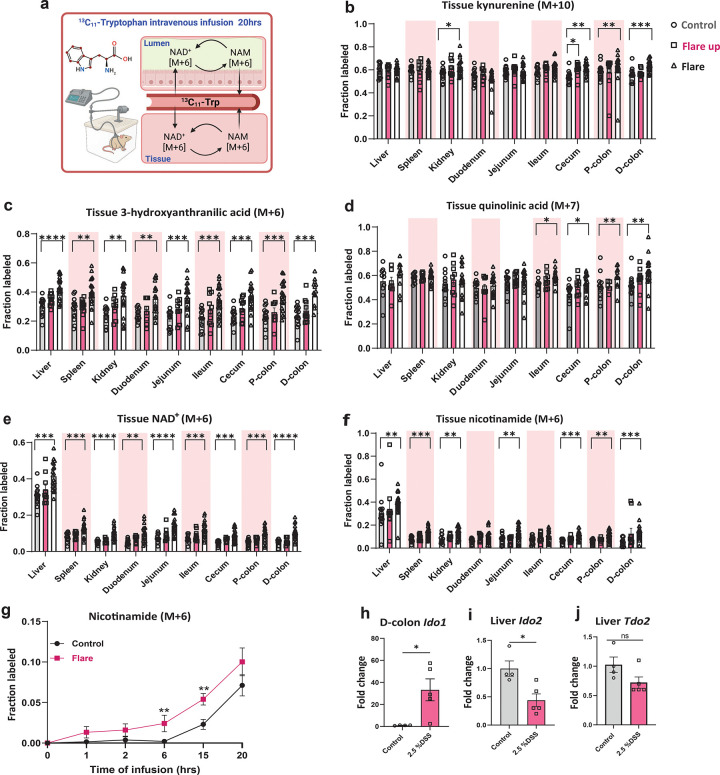
Enhanced NAD^+^ production from tryptophan during acute DSS-induced intestinal inflammation. **(a)** Experimental schematic of intravenous infusion of universally labeled-tryptophan (^13^C_11_-Trp) into pre-catheterized male C57BL/6 mice (11–12 weeks old) to assess NAD^+^ flux from tryptophan in host tissues and luminal samples. Tissue fractional labeling following 20-hour intravenous infusion of **(b)** kynurenine, **(c)** 3-hydroxyanthranilic acid, **(d)** quinolinic acid, **(e)** NAD^+^ and **(f)** nicotinamide. **(g)** Serum fractional labeling of nicotinamide over 20-hour intravenous infusion during the active flare phase of intestinal inflammation. mRNA expression of genes involved in *de novo* NAD^+^ synthesis from tryptophan via the kynurenine pathway was measured by qRT-PCR and normalized to TATA-box binding protein (*Tbp*) in **(h)** distal colon; indoleamine 2,3-dioxygenase 1(*Ido1*) **(i)** liver; indoleamine 2,3-dioxygenase 2 (*Ido2*) and tryptophan 2,3-dioxygenase (*Tdo2*), during the active flare phase (days 8 and 11) compared to untreated mice. Data are presented as mean ± SEM, (n=10–20) in **b-g** and (n=4–5) in **h-j**. Statistical significance was determined by Kruskal-Wallis test with post hoc Dunn’s test for comparisons among more than two groups and Mann-Whitney U test for two-groups comparisons. NS, not significant, § <0.1, *P<0.05, **P<0.01, ***P<0.001, and ****P<0.0001. (a) was created with Biorender.com

To assess whether the flux of Trp to NAD^+^ is altered by colitis induction, we performed *in vivo* isotope tracing using stable isotope-labeled Trp (U-^13^C_11_). The tracer was infused at a constant rate of 1.25 nmol/g body weight/min for 20 hrs in both DSS-treated and untreated mice during both the early flare up and active flare phases of inflammation (Figure 2a). In the serum, the fraction labeled Trp (M+11) and Kyn (M+10) were not significantly altered between DSS-treated and control mice at different phases of inflammation (Figure S2c–d). Moreover, the whole-body analysis of tissues revealed no difference in Trp (M+11) labeling between diseased and control mice (Figure S2e). However, there was an increase in the fraction labeled Kyn (M+10) in the cecum during the early flare up and in the kidney, cecum, and colon during the active flare phase, suggesting an enhanced flux of Trp via the KP in acute DSS-induced colitis (Figure 2b). This was further confirmed by increased labeling of the downstream metabolites with the expected label, 3-hydroxyanthranilic acid (3HAA, M+6) and QUIN (M+7) (5) (Figure 2c–d). Ultimately, KP-mediated Trp catabolism resulted in elevated NAD^+^ (M+6) production in multiple tissues during active flare phase, with the highest levels detected in the liver (Figure 2e). This elevation is consistent with the liver’s role as the primary site of Trp metabolism via the *de novo* pathway, where it synthesizes NAD^+^ from Trp and releases NAM (M+6) to support NAD^+^ synthesis in extrahepatic tissues (5). The release of NAM (M+6) from NAD^+^ (M+6), which can either be recycled to synthesize NAD^+^ or secreted into the circulation, was elevated in most tissues including the liver, spleen, kidney, small intestine and colon during the active flare phase (Figure 2f). Circulating NAM (M+6) levels increased at 6 hours in the active flare but not early flare up phase compared to control (Figure 2g and S2f).

The observed increase in Trp downstream metabolites supports the activation of the KP during intestinal inflammation, which has been previously described to be mediated by inflammatory cytokines and immune cell activation (30). Accordingly, colonic gene expression of *Ido1* was upregulated, reflecting enhanced local Trp catabolism during intestinal inflammation (Figure 2h). Interestingly, DSS treatment led to a significant downregulation of *Ido2*, along with reduced—but not statistically significant—*Tdo2* expression in the liver (Figure 2i). We observed the previously described metabolic blockade at the level of *Qprt* (Figure S2g–h). At first glance, the detection of increased tryptophan-derived NAD^+^ (M+6) flux appears paradoxical. However, because NAD^+^ (M+6) can also originate from nicotinamide (NAM, M+6) recycled through hepatic metabolism, our data suggest that the majority of NAD^+^ (M+6) detected in the colon arises from NAM salvage rather than *de novo* synthesis, which remains impaired due to the *Qprt* blockade.

Overall, these findings reveal tissue-specific rewiring of Trp catabolism in response to colitis, in which Trp fulfills a dual role: fueling *de novo* NAD^+^ synthesis primarily in the liver and supporting hepatic recycling of NAD^+^ to NAM to sustain systemic NAD^+^ homeostasis through the salvage pathway.

### Acute Colitis Enhances Microbial *de novo* NAD^+^ Synthesis from Tryptophan

**Fig.3|:**
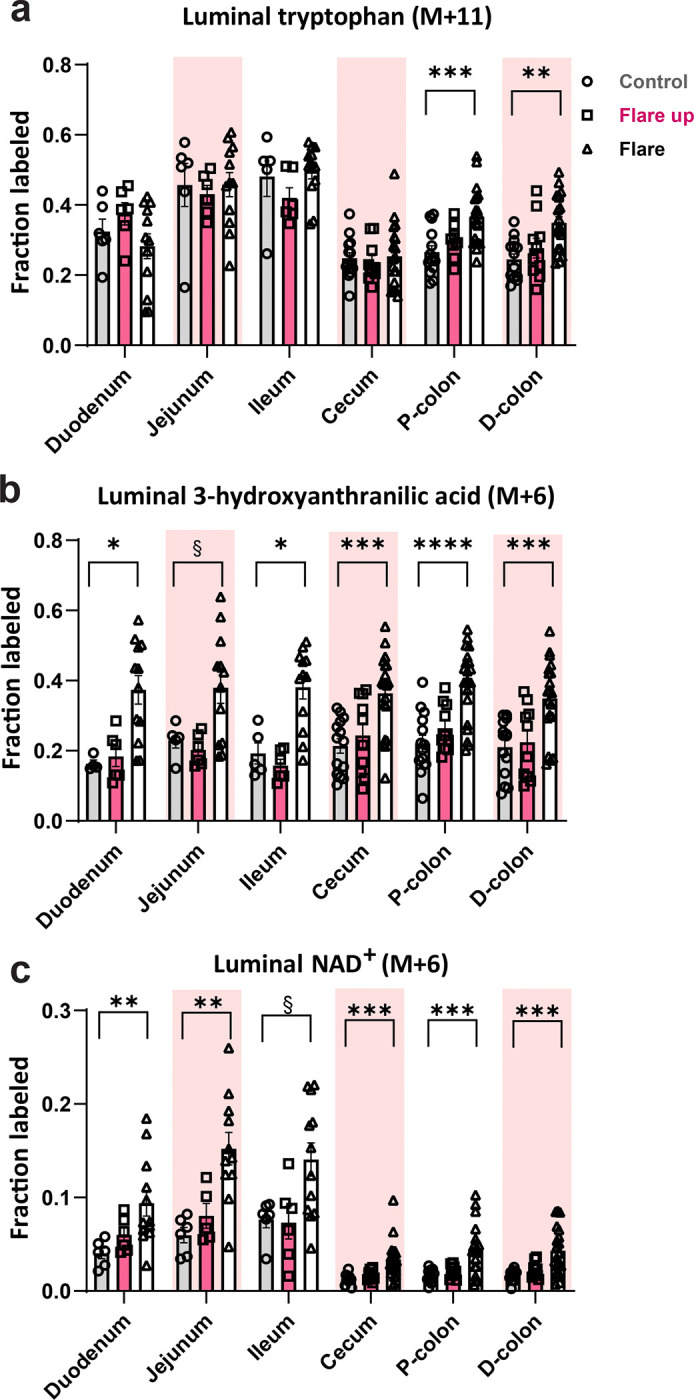
Elevated flux of tryptophan to luminal NAD^+^ during the active flare phase of acute DSS-induced colitis. LC-MS luminal fraction of metabolites labeled from the tryptophan tracer (shown in figure 2) following 20-hour intravenous infusion of **(a)** tryptophan, **(b)** 3-hydroxyanthranilic acid, and **(c)** NAD^+^. Data are presented as mean ± SEM, (n= 10–20). Statistical significance was determined by Kruskal-Wallis test with post hoc Dunn’s test for comparisons among more than two groups. NS, not significant, § <0.1, *P<0.05, **P<0.01,***P<0.001, and ****P<0.0001.

Previous reports highlighted the pivotal role of intestinal microbes in Trp metabolism. Many bacterial species possess enzymes that convert Trp into metabolites essential not only for bacterial functions but also for facilitating key communication pathways between the immune system and GI tract (31–33). Having established that flux of Trp to host NAD^+^ was impacted by intestinal inflammation, we next investigated whether intestinal inflammation similarly disrupts microbial NAD^+^ production in the gut lumen. We measured NAD^+^ levels in the luminal samples collected from different regions of the small intestine and colon, using the tracing approach described in Figure 2. We observed an increase of approximately 15% in Trp (M+11) labeling in the proximal and distal colon lumen during the active flare phase compared to control (Figure 3a). We noted a significant decrease of Kyn (M+10) in the D-colon during the active flare phase (Figure S3a). The fractional labeling of 3HAA (M+6) significantly increased in multiple luminal regions during the active flare phase compared to controls (Figure 3b), while the labeled fraction of QUIN (M+7) remained comparable between DSS-treated and control groups (Figure S3b). Flux of Trp to luminal NAD^+^ (M+6) increased in different luminal regions during the active flare phase compared to control (Figure 3c), with enhanced recycling of NAM (M+6) observed in the duodenum lumen during the active flare phase and in the ileum lumen during the early flare up phase (Figure S3c). These observations indicate a potentially significant contribution of the gut microbiome to Trp catabolism in acute colitis.

### Colitis-Induced Alterations of Tryptophan Metabolizing Bacteria at the Site of Inflammation

**Fig.4|:**
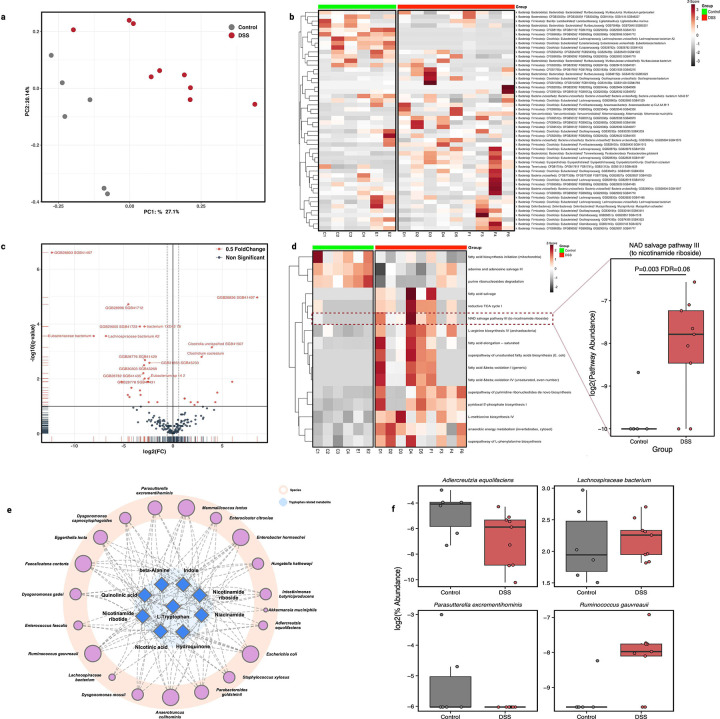
Integrative pathway and taxa analysis of the gut microbiota alterations in acute colitis **(a)** Bray–Curtis PCoA of species-level profiles shows clear separation between control (gray) and DSS (red) groups during the active phase of colitis (days 8 and 11) (PERMANOVA, R^2^ = 0.216, p = 0.002), indicating significant remodeling of the microbial functional potential during DSS-induced colitis. **(b)** Species-level heatmap of the top 50 most variable taxa, clustered by relative abundance Z-scores across samples. Hierarchical clustering reveals distinct community compositions between groups, with several *Clostridium* and *Lachnospiraceae* species enriched in DSS-treated mice during the active flare phase. **(c)** Volcano plot of differential species abundance between DSS and control groups. 33 species were significantly altered (FDR < 0.1, |log_2_FC| > 0.5), including 11 species enriched in DSS-treated group and 22 reduced relative to control. **(d)** Heatmap of significantly altered metabolic pathways (FDR<0.1). DSS-induced colitis group shows enrichment of lipid metabolism and NAD^+^ salvage pathway, and depletion of amino acid and purine biosynthetic pathways, suggesting a shift toward energy metabolism and stress-adaptive functions. **(e)** Predicted metabolite–taxon network linking 20 microbial taxa to 9 tryptophan-related metabolites via 110 high-confidence edges. **(f)** Altered tryptophan-metabolizing taxa including four differentially abundant species (*Lachnospiraceae* spp, *Adlercreutzia equolifaciens*, *Ruminococcus gauvreauii*, *Parabacteroides goldsteinii*), potentially modulating tryptophan metabolism during acute intestinal inflammation.

Having observed increased labeled Trp (M+11) in the colon lumen during the active flare phase, we next investigated whether DSS-induced colitis alters the abundance of Trp-metabolizing bacteria. Metagenomics profiling of the fecal contents revealed a distinct alteration in the gut microbiome composition (Figure 4A) and diversity (Figure S4a) between DSS-treated mice and control during the active flare phase. Beta diversity analysis of species-level community composition revealed a significant shift with clear stratification by treatment group (Figure 4a, PERMANOVA: R^2^ = 0.2168, p = 0.002). DSS-treatment was associated with a reduction in observed species, but not their distribution (Figure S4a). Differential abundance analysis between DSS-treated and vehicle controls identified 33 species (22 increased in control, 10 increased in DSS-treated; FDR ≤ 0.1; Figure 4b–c). To understand the metabolic impacts of these taxonomic shifts, we performed pathway analysis uncovering similar clustering patterns and uncovering 103 differentially abundant pathways (FDR ≤ 0.1, Figure S4b and Figure 4d). Differential pathways representing fatty acid biosynthesis and nucleotide metabolism were enriched in controls (Figure 4d). In line with the observed NAD^+^ metabolic rewiring (Figure 3), metagenomic data uncovered that DSS-treatment increased microbial NAD^+^ salvage pathway III generating NR suggesting microbial contributions to altered NAD^+^ precursor availability (Figure 4d). Owing to the poorly characterized nature of the mouse microbiome, to better understand the organisms which drove altered NAD metabolism, we integrated species abundances with Trp metabolites from metabolomics resulting in a predicted network of 389 metabolites, 20 taxa, and 4,070 edges. Zero-order filtering refined this to 9 metabolites and 20 taxa with 110 edges, highlighting species interacting with Trp and its related metabolites via well-annotated pathways (Figure 4e). The network analysis identified 20 bacterial species involved in Trp metabolism during DSS-induced colitis. Of those, 4 species, *Lachnospiraceae* spp., *Adlercreutzia equolifaciens, Ruminococcus gauvreauii, and Parabacteroides goldsteinii,* were significantly altered between DSS and control mice, suggesting a potential role in modulating Trp metabolism during the active flare phase of acute colitis (Figure 4f).

### Induction of Acute Colitis Drives Tissue-Specific Alterations of Tryptophan-Dependent Metabolites in the Host and Gut Microbiota

**Fig.5|:**
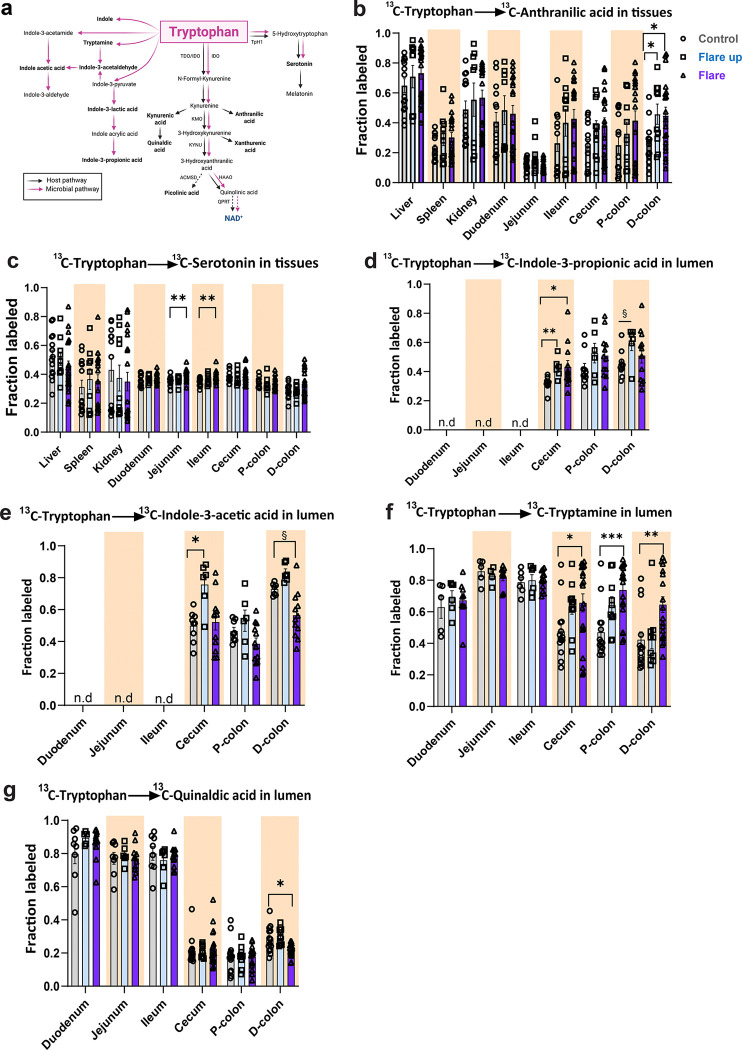
Acute colitis alters tryptophan-dependent pathways in the host tissues and gut microbes. **(a)** Overview of tryptophan metabolism in host tissues and gut microbiota. Metabolites shown in bold are compounds measured in the study. The fractional labeling of (^13^C_11_-tryptophan) over 20-hour intravenous infusion (as in figure 2), measured by LC-MS in different tissues for **(b)** anthranilic acid, **(c)** serotonin; and luminal regions of the gastrointestinal tract: **(d)** indole-propionic acid, **(e)** indole-acetic acid, **(f)** tryptamine, **(g)** quinaldic acid. Data are presented as mean ± SEM, (n= 10–20). Statistical significance was determined by Kruskal-Wallis test with post hoc Dunn’s test for comparisons among more than two groups. NS, not significant, § <0.1, *P<0.05, **P<0.01, ***P<0.001, and ****P<0.0001. (a) was created with Biorender.com

We next investigated whether the degradation of Trp through the KP affects the production of metabolites from other Trp-dependent pathways, which may, in turn, alter the gut homeostasis. Apart from its role in NAD^+^ biosynthesis, the KP produces several bioactive metabolites from Kyn with distinct neuroactive and immunomodulatory properties (Figure 5a) (25). Kynurenic acid (KynA) is produced from kynurenine (Kyn) by kynurenine aminotransferases (KAT I–IV), while anthranilic acid (AnthA) is generated from Kyn through the activity of kynureninase (KYNU). Similarly, xanthurenic acid (XanA) is formed from 3-hydroxykynurenine by KATs. In addition, picolinic acid (PicoA) is synthesized from 2-amino-3-carboxymuconate-6-semialdehyde (ACMS) through ACMS decarboxylase (ACMSD), which serves as a branch point that redirects the KP from production of QUIN and NAD^+^ (25).

In addition to the KP, Trp is also metabolized through the serotonin (5-hyroxytryptamine, 5-HT) pathway, in which serotonin serves as a key neurotransmitter involved in mood regulation, sleep, and gastrointestinal motility (25). To address this, we measured the fractional labeling of Trp-derived metabolites using Trp (U-^13^C_11_) (M+11), as described in Figure 2, in host tissues and gut lumen of DSS-treated and control mice at different stages of intestinal inflammation (Figure 5a).

Within host tissues, the fractional labeling of kynurenic acid (KynA) from Trp revealed a significant increase in the duodenum during the early flare up phase, however, no differences in the fractional labeling were observed across other tissues during the active flare phase (Figure S5a). Labeled anthranilic acid (AnthA) was not altered in different tissues, however, we detected increased flux of Trp to AnthA in the colon in both early flare up and active flare phases (Figure 5b). Flux of Trp to xanthurenic acid (XanA) and picolinic acid (PicoA) metabolites produced from the KP, were not altered during the active phase of DSS treatment (Figure S5b–c). In addition to the KP produced metabolites, the fraction labeled of serotonin was significantly increased in the jejunum and ileum during the active flare phase, suggesting enhanced flux of Trp to serotonin biosynthesis by enterochromaffin cells (EC) in the gut lining (34). However, flux of Trp to serotonin was not altered in other tissues including the colonic tissues (Figure 5c). We also quantified 5-methoxytryptophan (5-MTP) levels, a Trp-derived metabolite that may play a protective role in inflammation in preclinical studies (35–37). Acute intestinal inflammation had no detectable impact on the fractional labeling of 5-MTP in different tissues (Figure S5d). Additionally, the fractional labeling of quinaldic acid (QA), a Trp-derived metabolite produced from KynA, remained relatively stable in different tissues during different phases of intestinal inflammation (Figure S5e).

A growing body of evidence demonstrated that gut-microbiota derived indole and its derivatives produced in the intestine including indole-3-acetic acid (IAA), indole-3-propionic acid (IPA), and indole-3-lactic acid (ILA), significantly influence intestinal barrier function and immune responses (38–42). To this end, we investigated Trp degradation into microbial indole and its derivatives. No difference in the fractional labeling of Trp to indole was observed across different luminal regions, except for a slight decrease in the cecal lumen during the active flare phase compared to controls (Figure S5f). The fractional labeling of (ILA) and indole-3-acetaldehyde (IAAld) derived from Trp were not significantly altered in different luminal regions (Figure S5g–h). The fractional labeling of (IPA) significantly increased in the cecum during both phases of intestinal inflammation; in addition, a slight increase was also observed in the D-colon during the early flare up phase (Figure 5d). Microbial synthesis of (IAA) from Trp was reduced by approximately 15% (p=0.0754) in the D-colon during the active flare phase, while it increased in the cecal lumen during early flare up phase (Figure 5e).

Tryptamine is a key Trp-derived metabolite that plays a dual role in inflammation, acting protectively under homeostasis but potentially exacerbating inflammation during dysbiosis (43–45). We observed increased flux of Trp to tryptamine in the cecal and colon lumen during the active flare phase of inflammation, while flux in the small intestine remained unchanged, implying moderate effects of colitis on upper GI tract tryptamine production (Figure 5f).

Unlike in host tissues, our data showed no alterations in the production of serotonin across diseased conditions in the intestinal lumen (Figure S5i). However, flux from Trp to luminal KYNA increased in the ileum lumen during the active flare phase (Figure S5j). In addition, the fractional labeling of QA was reduced in the D-colon during the active flare phase, reflecting altered flux from Trp to QA during intestinal inflammation (Figure 5g). These data suggest that the systemic pool of Trp reaching the colon is utilized by the gut microbiota for metabolites production, with the generation of specific Trp-derived metabolites varying during the active phase of colitis. This is likely to reflect adaptive host-microbial responses aimed at maintaining gut homeostasis under inflammatory stress.

### Inflammation Rewires Tissue NAD^+^ Metabolism Through Salvage Pathway Activation

**Fig.6|:**
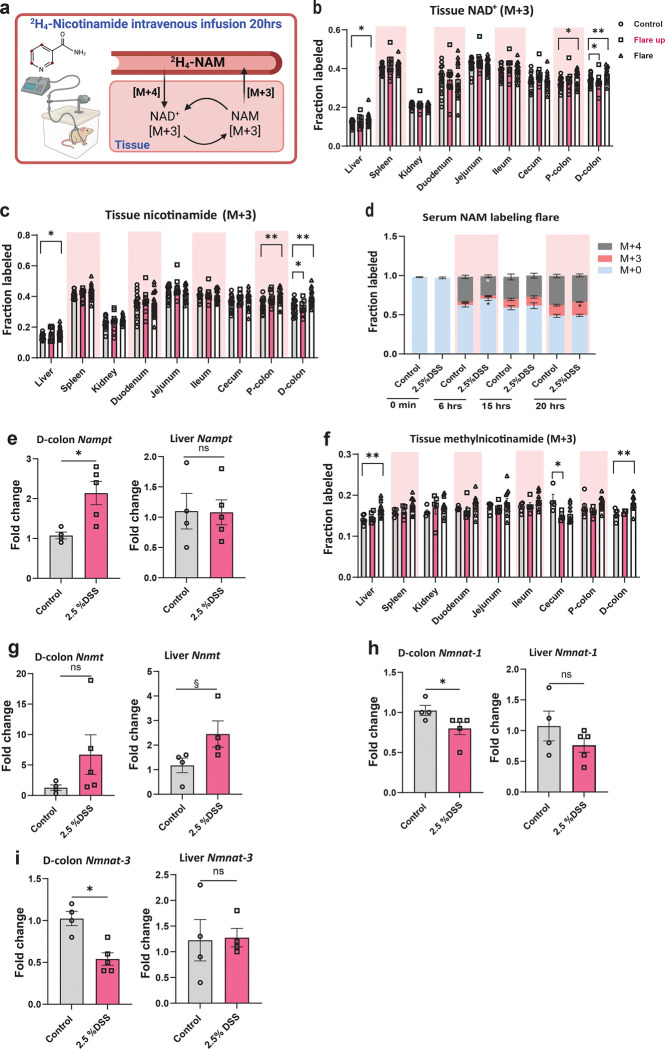
Inflammation enhances NAD^+^ production from nicotinamide via the salvage pathway in a tissue-specific manner. **(a)** Overview schematic of experimental setup for intravenous infusion of deuterium-labeled nicotinamide (2,4,5,6-^2^H_4_-NAM) into pre-catheterized male C57BL/6 mice (11–12 weeks old) to assess NAD^+^ flux from nicotinamide (NAM) via the salvage pathway in host tissues. Tissue fractional labeling following 20-hour intravenous infusion of **(b)** NAD^+^ and **(c)** nicotinamide. **(d)** Fractional labeling of nicotinamide in the serum post 20-hour intravenous infusion during the flare phase of intestinal inflammation (M+0: unlabeled NAM, M+3: recycled NAM, M+4: infusate NAM). **(e)** qRT-PCR analysis of mRNA expression normalized to *Tbp* in distal colon and liver tissues during the active flare phase (days 8 and 11) of nicotinamide phosphoribosyltransferase (*NAMPT*) compared to control. **(f)** Fractional labeling of methylnicotinamide in host tissues. qRT-PCR analysis of mRNA expression normalized to *Tbp* in distal colon and liver tissues during the active flare phase (days 8 and 11) compared to control for **(g)** nicotinamide N-methyltransferase (*NNMT*), **(h)** nicotinamide mononucleotide adenylyltransferase 1 (*NMNAT1*; nuclear), and **(i)** nicotinamide mononucleotide adenylyltransferase 3 (*NMNAT 3*; mitochondrial). Data are presented as mean ± SEM, in **b-d** and **f** (n=8–18), in **e,g,h** (n=4–5). Statistical significance was determined by Kruskal-Wallis test with post hoc Dunn’s test for comparisons among more than two groups and Mann-Whitney U test for two-groups comparisons. NS, not significant, § <0.1, *P<0.05, **P<0.01, ***P<0.001, and ****P<0.0001. (a) was created with Biorender.com

The salvage pathway is the major route of NAD^+^ production, balancing its continual consumption by NAD^+^ consuming enzymes and maintaining cellular NAD^+^ levels by recycling NAM back to NAD^+^ via the rate-limiting enzyme nicotinamide phosphoribosyltransferese (NAMPT). This pathway is significant for both normal physiological cellular function and disease states including IBD, where chronic inflammation imposes a high metabolic demand (46–49). To investigate whether the flux from NAM via the salvage pathway to NAD^+^ was impacted by acute colitis, we infused with (2,4,5,6-^2^H) NAM (M+4) at a constant rate of 0.1 nmol/g body weight/min in DSS-treated and control mice at different phases of intestinal inflammation (Figure 6a). The deuterated NAM (M+4) used in the experiment has a deuterium atom at the redox-active (4-^2^H) site (5, 50). This labeling remains with free NAM but is lost once incorporated into NAD^+^. As a result, the tracer NAM (M+4) forms NAD^+^ (M+3), and NAD^+^ breakdown releases NAM (M+3), which can be recycled back to synthesize NAD^+^ or secreted into the circulation (5, 50). The flux from NAM (M+4) to NAD^+^ (M+3) increased in the inflamed colon and liver during the active flare phase, along with elevated levels of recycled NAM (M+3), indicating higher NAD^+^ turnover in those tissues (Figure 6b–c). Circulating NAM (M+3) increased significantly during the active flare phase but not during the early flare up compared to control, reflecting increase NAD^+^ turnover during intestinal inflammation (Figure 6d and S6a).

We noted a significant two-fold upregulation of *Nampt* expression in the colon (p= 0.0238), but not in the liver, indicating increased salvage pathway activity at the site of inflammation during the active flare phase (Figure 6e). Additionally, we quantified methylnicotinamide (MeNAM), a methylated product of NAM catalyzed by the nicotinamide N-methyltransferase (NNMT) (51) and observed increased fractional labeling of MeNAM in the liver and colon during the active flare phase without a corresponding accumulation of the downstream metabolites N-Me-4PY and N-Me-6PY (Figure 6f and S6 b–c). This suggests enhanced NAM methylation, potentially to prevent excess NAM accumulation, which can inhibit sirtuins (52, 53). *Nnmt* expression exhibited a trend toward upregulation in the liver and colon during the active flare phase, however, not statistically significant (p= 0.057 and p= 0.11, respectively) (Figure 6g). We observed a significant downregulation of nuclear *Nmnat-1* and mitochondrial *Nmnat-3* in the colon, but not in the liver (Figure 6h–i), whereas the expression of cytoplasmic *Nmnat-2* remained unchanged in both tissues during the active flare phase compared to controls (Figure S6d). These observations suggest that the salvage pathway is selectively activated in metabolically stressed tissues during acute colitis. This likely represents a coordinated adaptive response to depleted NAD^+^ levels in the colon during colitis, boosting the use of NAM for NAD^+^ synthesis, suggesting a coordinated metabolic activation to support NAD^+^ replenishment under inflammatory stress.

### The Cycling of NAD^+^ Precursors between the Host Tissues and Gut Microbiota is Maintained Despite Active Inflammation

Given the role of NAD^+^ precursors exchange between the host tissues and gut microbiota, we next examined whether acute DSS-induced colitis disrupts the interconversion of NAD^+^ precursors between host tissues and the gut microbiota

**Fig.7|:**
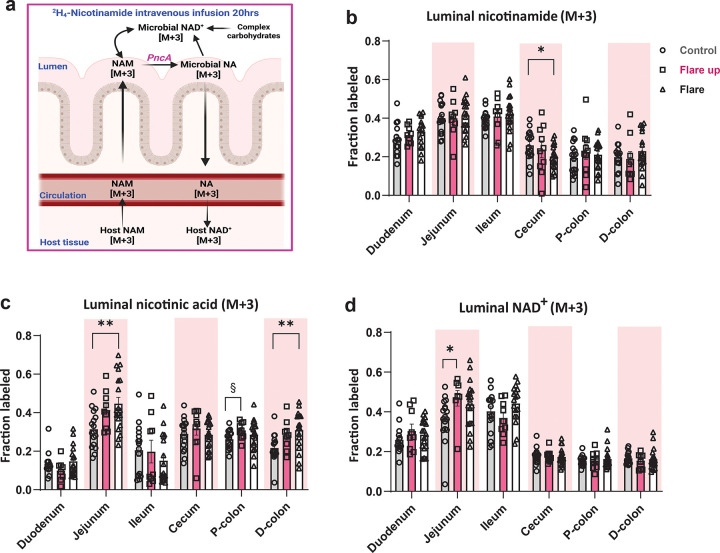
Acute intestinal inflammation does not compromise the metabolic cycling of NAD^+^ precursors between the host tissues and gut microbiota. **(a)** Schematic of the metabolic exchange of NAD^+^ precursors nicotinamide (NAM) and nicotinic acid (NA) between the host tissues and gut lumen. Host-derived NAM enters the gut lumen and contributes to microbial NAD^+^ either through conversion to NA and subsequently to NAD^+^, which supports both host and microbial NAD^+^ biosynthesis, or is directly converted to NAD^+^. Complex carbohydrates also contribute to microbial NAD^+^. LC-MS measurements of luminal microbial metabolites in the NAD^+^ salvage pathway after 20-hour intravenous infusion of the nicotinamide tracer (described in figure 6); (**b**) luminal nicotinamide, (**c**) luminal nicotinic acid, (**d**) luminal NAD^+^. Data are presented as mean ± SEM (n=8–18). Statistical significance was determined by Kruskal-Wallis test with post hoc Dunn’s test for comparisons among more than two groups. § <0.1, *P<0.05, **P<0.01, ***P<0.001, and ****P<0.0001. (**a**) was created with Biorender.com

Production of luminal NAD^+^ can occur via multiple routes, (1) host-derived NAM to microbial NA to NAD^+^, a major route, (2) host-derived NAM to NAD^+^, (3) or through complex carbohydrates that support *de novo* synthesis of NAD^+^ (7) (Figure 7a). We quantified the labeling patterns of luminal NAM, NA, and NAD^+^ from infused NAM (M+4), using the tracer described in Figure 6a. The host-derived NAM (M+3) was detected in the lumen all along the GI tract during intestinal inflammation, although it slightly decreased in the cecal lumen during the active flare phase compared to control (Figure 7b). Labeled microbial NA (M+3), generated from NAM (M+3), increased in the lumen of the jejunum and D-colon in the active flare phase, indicating enhanced flux from NAM (M+3) to microbial NA (M+3) to support NAD^+^ synthesis in the host tissues and gut microbiota (Figure 7c). NAM (M+4) was not detected in the luminal samples, supporting the notion that luminal NAM (M+3) is primarily derived from host tissue into the gut lumen. Luminal NAD^+^ (M+3) production from the shared precursors NAM and NA was comparable in DSS-treated and control mice in both phases, with a slight increase in the jejunum during the early flare up phase (Figure 7d). Labeled NA (M+3) was detected in the small intestine and colon, indicating the uptake of microbial NA (M+3) by the host tissues and reflecting the dynamic interactions between the gut microbiota and host tissues (Figure S7a). The gene expression of nicotinate phosphoribosyltransferase (*Naprt*) was comparable between DSS-treated mice and control in the colonic tissues, supporting the observed uptake of microbial NA by the host tissues (Figure S7b). Together, these data suggest that the cycling of NAD^+^ precursors is maintained, highlighting the resilience of the host and gut microbiota interactions during physiological stress, such as acute DSS-induced colitis.

### Tryptophan Catabolism during Acute Colitis Induction Leads to a Systemic Metabolic Rewiring to Replenish NAD^+^

**Fig.8|:**
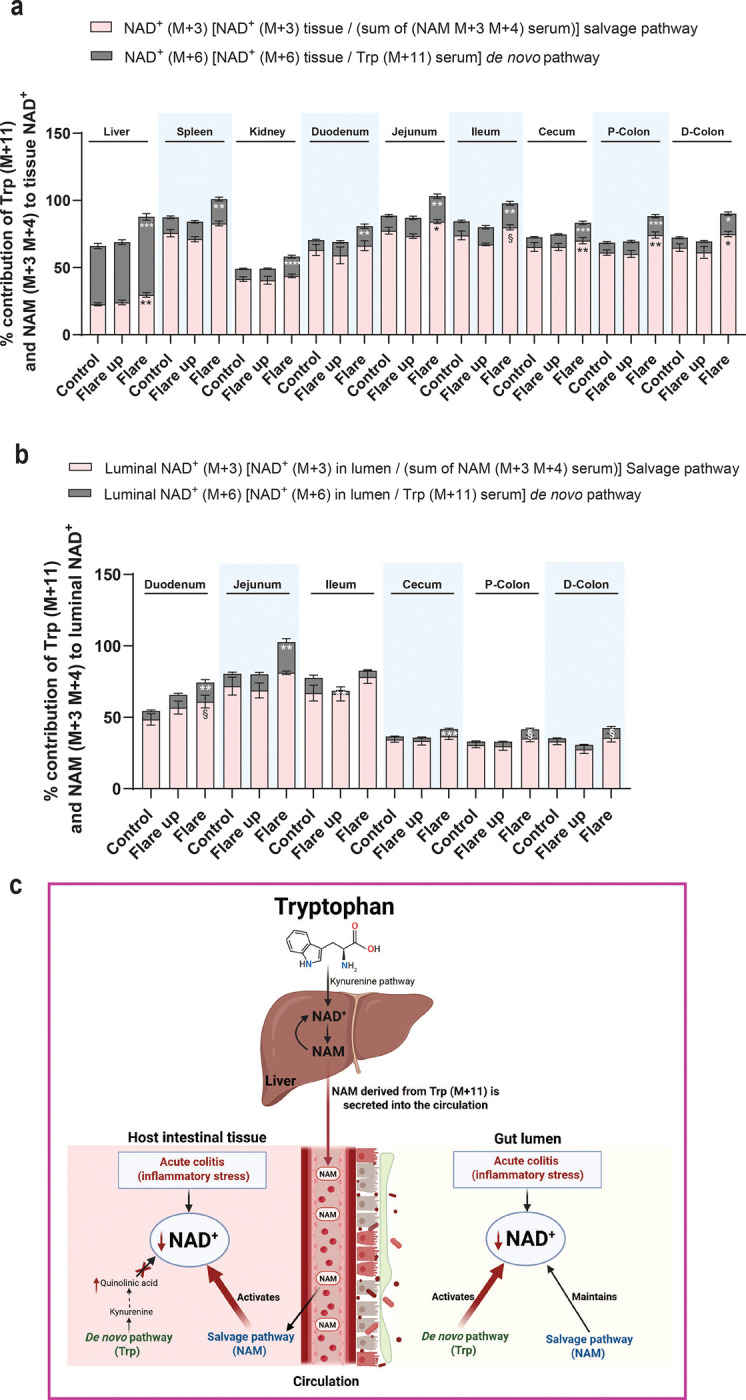
Acute colitis triggers systemic metabolic adaptation to restore NAD^+^ levels through activation of the salvage pathway. **(a)** Percent contributions of tryptophan and nicotinamide to (**a**) host NAD^+^ levels in different tissues and (**b**) luminal NAD^+^, across different phases of intestinal inflammation. In (**a**) and (**b**), the contribution of nicotinamide (M+4) to NAD^+^ (M+3) was calculated as [fraction of labeled NAD^+^ (M+3)/ the sum of (NAM+3 and NAM+4) in serum]. Tryptophan (M+11) contribution to tissue NAD^+^ (M+6) was quantified as [fraction of labeled NAD^+^ (M+6)/ fraction of labeled Trp (M+11) in the serum), following 20-hour intravenous infusion of the labeled-NAD^+^ precursors (depicted in figure 2 and 6). (**c**) Summary of the compensatory metabolic adaptation of host and microbial NAD^+^ metabolism during experimental acute colitis. Data are presented as mean ± SEM. Statistical significance was determined by Kruskal-Wallis test with post hoc Dunn’s test for comparisons among more than two groups. § <0.1, *P<0.05, **P<0.01, ***P<0.001, and ****P<0.0001. (c) was created with Biorender.com

Given the observed decline in NAD^+^ levels within colonic tissues and luminal contents, alongside compensatory increases in NAD^+^ production across other tissues during the acute colitis active flare phase, we next sought to determine the relative contributions of Trp and NAM to NAD^+^ pools in host tissues and the intestinal lumen. The analysis revealed that the liver relies on Trp for NAD^+^ production. During the active flare phase, a significant increase in Trp-derived NAD^+^ was observed in the spleen, kidney, small intestine, and colon. However, these tissues primarily depend on the salvage pathway for NAD^+^ production—using NAM directly or indirectly through recycled NAM (M+6) generated from tryptophan-derived NAD^+^. In colonic tissues, this recycling provides an alternative route to bypass the blockade in *de novo* NAD^+^ synthesis from Trp (5, 50, 54) (Figure 8a). The analysis of luminal NAD^+^ indicated that under physiological conditions, the salvage pathway from NAM is the primary source of luminal NAD^+^. However, during the active flare phase, there was a significant increase in the contribution of Trp to luminal NAD^+^ in the duodenum, jejunum, and cecum, accompanied by a significant decrease in NAD^+^ production from Trp in the ileum lumen during the early flare up phase. Additionally, a modest upward trend in Trp-derived luminal NAD^+^ was observed in the colon lumen (Figure 8b). The expression profiling of NAD^+^-consuming enzymes indicated no significant differences between the DSS-treated and control groups, except for CD38, which showed a modest increase, though not statistically significant, in both the colon and liver during the active flare phase (Figure S8a).

In host tissues, activation of the NAM–dependent salvage pathway provides a rapid, tissue-specific metabolic response to replenish depleted NAD^+^ levels under inflammatory stress. In parallel, the gut microbiota sustains NAD^+^ production primarily through the salvage pathway, while *de novo* synthesis contributes to microbially derived NAD^+^ during acute DSS-induced inflammation (Figure 8C). Collectively, these findings demonstrate that acute intestinal inflammation reshapes NAD^+^ metabolism in both host tissues and the gut microbiota. Coordinated activation of the *de novo* and salvage pathways is essential to maintain NAD^+^ levels that support energy metabolism, immune regulation, and intestinal homeostasis during acute colitis.

## Discussion:

Although previous studies have reported alterations in NAD^+^ levels in IBD, it remains unclear whether the imbalance is due to changes in NAD^+^ biosynthesis, consumption, or both (8, 9, 55, 56). Dysregulated NAD^+^ levels disrupt cellular metabolism and homeostasis, driving a cascade of physiological dysfunction. Impaired NAD^+^ availability can disturb energy metabolism, hinder cellular repair mechanisms, and escalate oxidative stress, which may exacerbate inflammatory processes in IBD (57–60).

In acute DSS-induced colitis, we observed a pronounced reduction of NAD^+^ levels in the inflamed colon and adjacent lumen. This decline results from two converging mechanisms. First, as we previously reported, *de novo* NAD^+^ synthesis from Trp is impaired in the inflamed mucosa due to a metabolic bottleneck at *Qprt* (54). Second, we show that an elevated trend of NAD^+^ consumption by colonic CD38 further contributes to this reduction, as NAD^+^ utilization outpaces its synthesis in response to heightened cellular energy demand. In compensation, acute colitis induces extensive metabolic reprogramming, with distinct NAD^+^ biosynthetic pathways activated across tissues according to their specific metabolic needs during inflammatory stress. The increased flux of Trp into NAD^+^ across multiple tissues underscores a systemic adaptive response mediated by activation of the KP, likely driven by proinflammatory cytokine signaling and immune activation (61–63). Although *de novo* synthesis remains obstructed at *Qprt* in the colon, Trp continues to serve as a key precursor for NAD^+^ production in the liver, where recycling of NAD^+^ to NAM enables redistribution of NAM to the colon to sustain NAD^+^ regeneration through the salvage pathway. The liver and colon thus exhibit distinct yet complementary NAD^+^ biosynthetic responses to inflammation, with enhanced flux through the salvage pathway in both tissues supporting the maintenance of NAD^+^ homeostasis under acute inflammatory conditions.

The enhancement of both the KP and salvage pathway during acute colitis suggests a compensatory mechanism to regenerate depleted NAD^+^ levels in the inflamed mucosa, thereby meeting the increased NAD^+^ demand associated with inflammation. This aligns with previous studies showing that inflammatory cytokines can drive metabolic changes to support immune responses, genomic stability, and tissue repair (64, 65).

An important aspect of our findings is the role of the gut microbiome in regulating NAD^+^ metabolism during inflammation. The increased flux of Trp to NAD^+^ in luminal regions suggests that microbial metabolism substantially contributes to local NAD^+^ production under inflammatory conditions. This highlights the dynamic interplay between host and microbial metabolism, where microbial activity can shape host tissue responses and influence energy and immune balance during inflammatory stress (66, 67). Moreover, given the microbiome’s capacity to synthesize NAD^+^ from precursors such as Trp and NAM (7), microbially derived NAD^+^ and related metabolites likely support the systemic NAD^+^ pool, reinforcing host metabolic resilience and modulating inflammatory responses in the context of IBD (68, 69).

The observed increase in labeled Trp in the colonic lumen may reflect alterations in bacterial Trp catabolism during acute inflammation. Our data reveal a shift of certain Trp-metabolizing bacteria, which may alter the host-microbiome dynamic and impact the gut homeostasis. Future work should further investigate the potential role of the altered gut bacteria involved in Trp metabolism, deciphering how they affect systemic metabolism and impact disease progression in chronic colitis.

Given the importance of NAD^+^ in immune function and tissue repair (70), modulating NAD^+^ metabolism may represent a promising therapeutic strategy for IBD. Our multi-omics approach provides a comprehensive overview of NAD^+^ metabolism in IBD, revealing important insights into tissue-specific metabolic responses and the role of the gut microbiota. Targeting key NAD^+^ biosynthesis pathways, particularly the salvage pathway, could help restore NAD^+^ homeostasis and mitigate inflammation in the gut (49). In line with this concept, we are currently evaluating an ileocolonic-release formulation of oral NAM for the treatment of mild to moderate ulcerative colitis (Ornatus 1, NCT06488625). Future studies should aim to dissect the contributions of individual microbial species to NAD^+^ production and explore how these interactions affect host health in other inflammatory diseases.

In summary, our study reveals that acute colitis induces a complex metabolic response, where distinct NAD^+^ biosynthesis pathways are activated in different tissues to meet the metabolic demands of inflammation. By integrating isotope tracing and multi-omics techniques, we have provided new insights into the role of NAD^+^ metabolism in IBD and highlighted potential therapeutic targets for restoring metabolic homeostasis in inflammatory diseases.

## Methods

### Animals

11–12-week-old male C57BL/6 (WT) mice pre-catheterized on the right jugular vein were purchased from Charles River Laboratories (Wilmington, MA). Animals were single-housed in a temperature-controlled facility and maintained on 12-hour-light-dark cycle (7AM-7PM). All mice were given ad libitum access to a normal chow diet (catalog# 5053, LabDiet) and acclimated for at least 7 days before experimental use. Animal studies were conducted at Pennsylvania State University and approved by the Institutional Animal Care and Use Committee (PROTO202202188).

### Acute DSS colitis induction in mice

Mice were randomly assigned into 2 groups (n= 8–20 group). The control group received normal drinking water without DSS, and the treated group was administered dextran sulfate sodium (DSS) (MW 36–50kDa, MP Biomedicals, Solon, OH) at a concentration of 2.5% (w/v) in drinking water for 5 days (17, 18). Following the initial treatment period (5 days), both groups received normal drinking water until the end of the experiment on day 11. The early flare up phase is characterized by mild colitis occurring on days 4–5, and the active flare phase spans from days 7 to 11, in which the colon is fully inflamed (19–21). During the experiment, mice were weighed daily, and fecal and serum samples were collected on days (1,3,5,8,11) and stored at −80°C. At the end of the experiment, mice were sacrificed by cervical dislocation, tissues and luminal samples were collected and snap frozen in liquid nitrogen.

### Clinical assessment of colitis severity

The severity of colitis was assessed by evaluating the following parameters daily: weight loss (0 points = no weight loss or gain, 1 point = 1–5% weight loss, 2 points = 6–10% weight loss, 3 points = 11–19% weight loss, 4 points = 20–25% weight loss, 5 points = >25% weight loss); stool consistency (0 points = normal and firm, 1 point = very slight change, 2 points = slight change and soft, 3 points = moderate change, 4 points = noticeable change, diarrhea, 5 points = severe change, runny diarrhea; bleeding stool (0 points = normal, 1 point = redness of perianal region, 2 points = slightly blood-streaked stool, 3 points = blood-streaked stool, 4 points = marked blood contamination, 5 points = bloody stool); posture (0 points = normal, 1 points = very slight change, 2 points = slightly curved, 3 points = moderate change, 4 points = strongly curved, 5 points = severe change and consistently curved); activity (0 point = normal active, 1 point = very slight change in activity, 2 points = reduced movement and clinging onto the cage, 3 points = moderate change, 4 points = noticeable change and rarely clinging onto the cage, 5 points = severe change, sitting still and no movement); fur (0 points = normal, 1 point = very slight change, 2 points = slightly dirty and scruffy, 3 points = moderate change, 4 points = noticeable change, dirty, and scruffy, 5 points = severe change, very dirty, dull, and scruffy). The DAI was calculated by combining scores of the measured parameters (18, 71)

### Histological analysis of disease activity

Postmortem, colonic tissues were excised and cut open longitudinally. The colon was rolled up as Swiss rolls from the distal to the proximal part and fixed in 10 % formalin. Paraffin sections were cut and stained with hematoxylin and eosin (H&E). Histological scoring displays the combined score of inflammatory cell infiltration and tissue damage as described elsewhere and was performed in a blinded fashion, as described previously (72)

### Murine Immunohistochemistry

Formalin-fixed paraffin-embedded colon section slides were deparaffinized in Xylene-substitute (Carl Roth GmbH + Co. KG, Karlsruhe, Germany) and rehydrated in ethanol (Th. Geyer GmbH & Co. KG, Hamburg, Germany). Antigen retrieval was carried out by heating the slides in citrate buffer (10mM, PH 6.0, prepared in the laboratory) for 20 minutes. The sections were submerged in 3% hydrogen peroxide (Sigma-Aldrich, Merck KGaA, Darmstadt, Germany) for 10 minutes to block endogenous peroxidases, and nonspecific binding was blocked using 5% BSA (Carl Roth GmbH + Co. KG, Karlsruhe, Germany) in PBS (Life Technologies GmbH, Darmstadt, Germany) for 1 hour. Tissues were incubated with the primary antibody Qprt (1:75, Biorbyt, orb317756) at 4°C overnight followed by a 45-minute incubation with a biotinylated secondary antibody (goat anti-rabbit IgG ready-to-use, Abcam, ab64256). Signal detection was performed using the Vectastain ABC Kit (Vector Laboratories, Peterborough, UK) according to the manufacturer’s instructions. Color development was carried out using the DAB Peroxidase Substate Kit (Vector Laboratories, Peterborough, UK). Tissue sections were then counterstained by hematoxylin (Sigma-Aldrich, Merck KGaA, Darmstadt, Germany), dehydrated in ethanol (Th. Geyer GmbH & Co. KG, Hamburg, Germany) and mounted with ROTI-Histokitt mounting medium (Carl Roth GmbH + Co. KG, Karlsruhe, Germany).

To quantify immunohistochemical (IHC) staining intensity, ten images per Swiss roll were acquired using Axio Observer A1 brightfield microscope (Zeiss, Germany) at 40x magnification with ZEN software under identical imaging conditions (exposure time, white balance, and color calibration). Images were analyzed in ImageJ version 1.54g using the IHC Toolbox plugin (https://imagej.net/ij/plugins/ihc-toolbox/ ) with the H-DAB model to isolate the brown signal corresponding to the secondary antibody. The resulting images were converted to 8-bit grayscale and thresholded (0–120) prior to quantitative analysis to determine the stained area and mean gray value. Staining intensity was calculated as the product of mean gray value and stained area. For each animal, values from technical replicates were averaged to obtain a single mean value.

### Enzyme-linked immunosorbent assay (ELISA)

Quantification of fecal Lipocalin-2 was used to assess intestinal inflammation. Briefly, fresh or frozen fecal samples from control and DSS-treated (active flare phase) mice were reconstituted in PBS containing 0.1% Tween 20 (100mg/ml) and vortexed for 20 minutes to create a homogenous suspension. The samples were then centrifuged at 12,000 rpm for 10 minutes at 4°C. Clear supernatants were collected and stored at −20°C until analysis. Lcn-2 levels in the supernatants were measured using ELISA kit purchased from (R&D systems DY1857, Minneapolis, MN, USA) following manufacturer’s instructions.

### RNA isolation and quantitative real time-polymerase chain reaction (qRT-PCR)

Total RNA was extracted from liver and colon tissues using TRIzol reagent (Thermo Fisher Scientific, Carlsbad, CA, USA) per the manufacturer’s protocol. The purified RNA 1.0 ug was reverse transcribed into cDNA using qScript cDNA synthesis kit (Quanta Biosciences, Beverly, MA, USA). Quantitative RT-PCR was performed using the PerfeCTa qPCR FastMix, UNG, Low ROX (Quanta Biosciences, Beverly, MA, USA) in a 20 ul reaction mixture containing cDNA and TaqMan probes (Thermo Fisher Scientific) per the manufacturer’s instructions for TaqMan assays. The cycling parameters were 95.0 C for 3 min, followed by 40 cycles of 95.0 C for 15 s, and 60C for 1 min. Relative quantification of each gene was calculated using 2^−ΔΔCt^, and normalized to TBP expression to yield a fold-change. A list of the primers used in the study is provided in supplement table 1.

### Intravenous infusion of mice

*In vivo* infusion of stable isotope labeled NAD precursors, [2,4,5,6-^2^H]-NAM and [U-^13^C_11_]-Trp (Cambridge Isotope Laboratories, Andover, MA, USA), were infused separately in control and DSS-treated mice at different stages of intestinal inflammation (early flare up phase= day 3–4 post colitis induction; active flare phase= day 7–8, and day 10–11) for 20 hours to achieve steady state NAD^+^ labeling from labeled precursors in different tissues. The mouse infusion setup included a tether and swivel system (Instech Laboratories, Plymouth Meeting, PA) to allow free movement of the mouse in the cage with bedding materials and access to food and hydrogel water (Clear H_2_O, Portland, ME). Isotope labeled NAD^+^ precursors were prepared as a solution in 0.9% NaCl (50mM for [U-^13^C_11_]-Trp, and 4mM for [2,4,5,6-^2^H]-NAM) and infused via the catheter at a constant rate of 0.5μl per 20 g body weight per min. Blood samples (~ 20μl) were collected via tail bleeding using microvette CB 300 CAT blood collection tubes (REF 16.440.100, SARSTEDT AG& Co.KG, Nümbrecht, Germany) at different time points (0min, 15min, 30min, 1hr, 2hr, 6hr, 15hr, and 20hr) and centrifuged at 16,000 g for 25 minutes at 4°C to separate serum. At the end of the infusion, mice were euthanized by cervical dislocation. Tissues and luminal samples were dissected and separated and immediately clamped with a pre-cooled Wollenberger clamp in foil and stored in liquid nitrogen. Tissues, serum, lumen, fecal samples were kept at −80°C prior metabolites extraction for mass spectrometry analysis (50).

### Metabolite extraction from serum, tissues, and lumen

Serum was thawed on ice before extracting with −20°C 100% methanol with a volume of 65μl per 5μl serum, vortexed for 15 seconds, incubated on dry ice for 10 minutes, and centrifuged at 16,000 g for 25 minutes. The supernatant was transferred into MS vials (Thermo Scientific, Rockwood, TN, USA) for LC-MS analysis. Tissues, lumen, and fecal samples were weighed (~20mg), homogenized with liquid nitrogen in a cryomill (Retsch) at 25Hz for 45 seconds, before extracting with 40:40:20 acetonitrile: methanol: water, with a volume of 40μl solvent per 1mg of sample. The samples were vortexed for 10 seconds, transferred to new 2mL Eppendorf tube, incubated on ice for 10 minutes, and centrifuged at 16,000 g for 30 minutes. The supernatants transferred to new Eppendorf tubes and centrifuged at 16,000 g for 30 minutes. The top 50μl of the supernatant was used for LC-MS analysis.

### Metabolite measurement

Extracts were analyzed within 24 hours by liquid chromatography coupled to a mass spectrometer (LC-MS). The LC–MS method was based on hydrophilic interaction chromatography (HILIC) coupled to the Orbitrap Exploris 240 mass spectrometer (Thermo Scientific) (Wang, 2019). The LC separation was performed on a XBridge BEH Amide column (2.1 × 150 mm, 3.5 *μ*m particle size, Waters, Milford, MA). Solvent A is 95%: 5% H2O: acetonitrile with 20 mM ammonium acetate and 20mM ammonium hydroxide, and solvent B is 90%: 10% acetonitrile: H2O with 20 mM ammonium acetate and 20mM ammonium hydroxide. The gradient was 0 min, 90% B; 2 min, 90% B; 3 min, 75% B; 5 min, 75% B; 6 min, 75% B; 7 min, 75% B; 8 min, 70% B; 9 min, 70% B; 10 min, 50% B; 12 min, 50% B; 13 min, 25% B; 14min, 25% B; 16 min, 0% B; 18 min, 0% B; 20 min, 0% B; 21 min, 90% B; 25 min, 90% B. The following parameters were maintained during the LC analysis: flow rate 150 mL/min, column temperature 25 °C, injection volume 5 μL and autosampler temperature was 5 °C. For the detection of metabolites, the mass spectrometer was operated in both negative and positive ion mode. The following parameters were maintained during the MS analysis: resolution of 180,000 at m/z 200, automatic gain control (AGC) target at 3e6, maximum injection time of 30 ms and scan range of m/z 70–1000. Raw LC/MS data were converted to mzML format using the command line “msconvert” utility. Data was analyzed via EL-MAVEN software version 12. All isotope labeling patterns were corrected for natural abundance ^13^C and ^2^H using AccuCor (73)

### Metagenomic DNA extraction

Mice fecal samples from control and DSS-treated groups (flare phase) selected for metagenomic sequencing were extracted using the DNeasy PowerSoil Kit (Qiagen, Germantown, MD) following the standard protocol in the One Health Microbiome Center Co-Laboratory at Pennsylvania State University. Detailed protocol can be found at https://github.com/BisanzLab/OHMC_Colaboratory. Extraction blanks were included to assess environmental and reagent contamination. Briefly, fecal samples were weighed (~50 mg) and homogenized after bead beating for 5 minutes at 25 Hz using Qiagen Tissuelyzer III (Qiagen, Germantown, MD). Protein and solid particles were precipitated, and genome DNA was further purified on provided columns. Purified genomic DNA was eluted in nuclease-free H_2_O. Genomic DNA was quantified by spectrophotometry (Nanodrop One) and shipped to Novogene (Sacramento, CA) on dry ice for library preparation and sequencing via NovaSeq X with PE150 reads. Samples were sequenced with an 4.5±1.9 Gbases of sequencing data (mean ± sd).

### Metagenomic sequencing and data processing

Samples were processed by first using FastP to detect and remove adapters, remove polyG runs, and perform sliding window quality filtering (74). Next reads mapping to host DNA were removed using Kneaddata using provided reference assemblies (75). Sample normalization factors were determined using MicrobeCensus (76). Taxonomic abundances were determined using MetaPhlAn v4.0.6 including viruses and unclassified estimation. Gene family abundances and pathway abundances were determined using HUMAnN v3.9 against the uniref90 database (77). Taxonomic and Pathway abundances were normalized as the log2 of the reported abundance. Gene family abundances were normalized as RPKG (read per kilobase per genome equivalent as derived from MicrobeCensus). Alpha diversity metrics were determined using Vegan v2.6–10. Principal coordinates analysis was performed as implemented in Ape v5.8–1. Statistical analysis was performed log-transformed data and Welch’s t-test. PerMANOVA was implemented using the adonis2 function of Vegan with 999 permutations. All P-values were corrected with Benjamini Hochberg False Discovery Rate unless otherwise indicated.

### Integrative multi-omics network analysis of tryptophan metabolism

To identify microbial taxa capable of metabolizing tryptophan and its derivatives, we used an integrative knowledge-based multiomics integration approach using the OmicsNet 2.0 web interface (78).Tryptophan-related metabolites captured through targeted metabolomics analysis and MetaPhlAn-derived species-abundances were used as an input for the network construction via logistic regression models trained on high-quality genome-scale metabolic models (GEMs). EMBL GEM repository were used to construct an initial metabolite–taxon interaction network, with confidence threshold of ≥ 0.7 and excluding metabolites without defined pathway annotations (e.g., KEGG or MetaCyc). The resulting global network was converted to a zero-order network to retain only species and metabolites that were part of the original input seed set.

### Statistical analysis

Statistical analysis was performed using GraphPad PRISM software version 10.4.0 (GraphPad Software, La Jolla, CA, USA). Unless otherwise stated, all data are presented as the mean ± SEM. Comparisons between two groups were performed using Mann-Whitney U test. For comparison between more than two groups, Kruskal-Wallis test followed by Dunn’s multiple comparisons was applied. Statistical significance was defined as (§ <0.1, *P<0.05, **P<0.01, ***P<0.001, and ****P<0.0001). N represents the number of biological samples from two independent experiments.

## Supplementary Material

Supplement 1

## Abbreviations

AnthA: Anthranilic acid
5-MTP: 5-methoxytryptohan
3-HAA: 3-Hydroxyanthranilic acid
DC: Distal colon
DDS: Dextran sulfate sodium
IBD: Inflammatory bowel disease
IAA: Indole-3-acetic acid
ILA: Indole-3-lactic acid
IPA: Indole-3-propionic acid
IDO1/2: Indoleamine 2,3-dioxygenase 1/2
Kyn: Kynurenine
KP: Kynurenine pathway
KynA: Kynurenic acid
MeNAM: Methylnicotinamide
NAD^+^: Nicotinamide adenine dinucleotide
NAM: Nicotinamide
NA: Nicotinic acid
NAMPT: Nicotinamide phosphoribosyltransferase
NAPRT: Nicotinate phosphoribosyltransferase
NMNAT1–3: Nicotinamide-nucleotide adenylyltransferase 1–3
PC: Proximal colon
PicoA: Picolinic acid
QUIN: Quinolinic acid
QA: Quinaldic acid
Trp: Tryptophan
TOD: Tryptophan 2,3-dioxygenase
XanA: Xanthurenic acid
