# Supplementary material for: Tracing NAD+ metabolism uncovers adaptive coordination between host and microbiome during colitis
Source: Res Sq. 2025 Dec 4:rs.3.rs-8195970. Preprint. [Version 1] doi: 10.21203/rs.3.rs-8195970/v1 (PMC12687811; doi:10.21203/rs.3.rs-8195970/v1)
Supplement: Supplement 1 [file NIHPPrs8195970v1-supplement-1.pdf]

Fig.S1

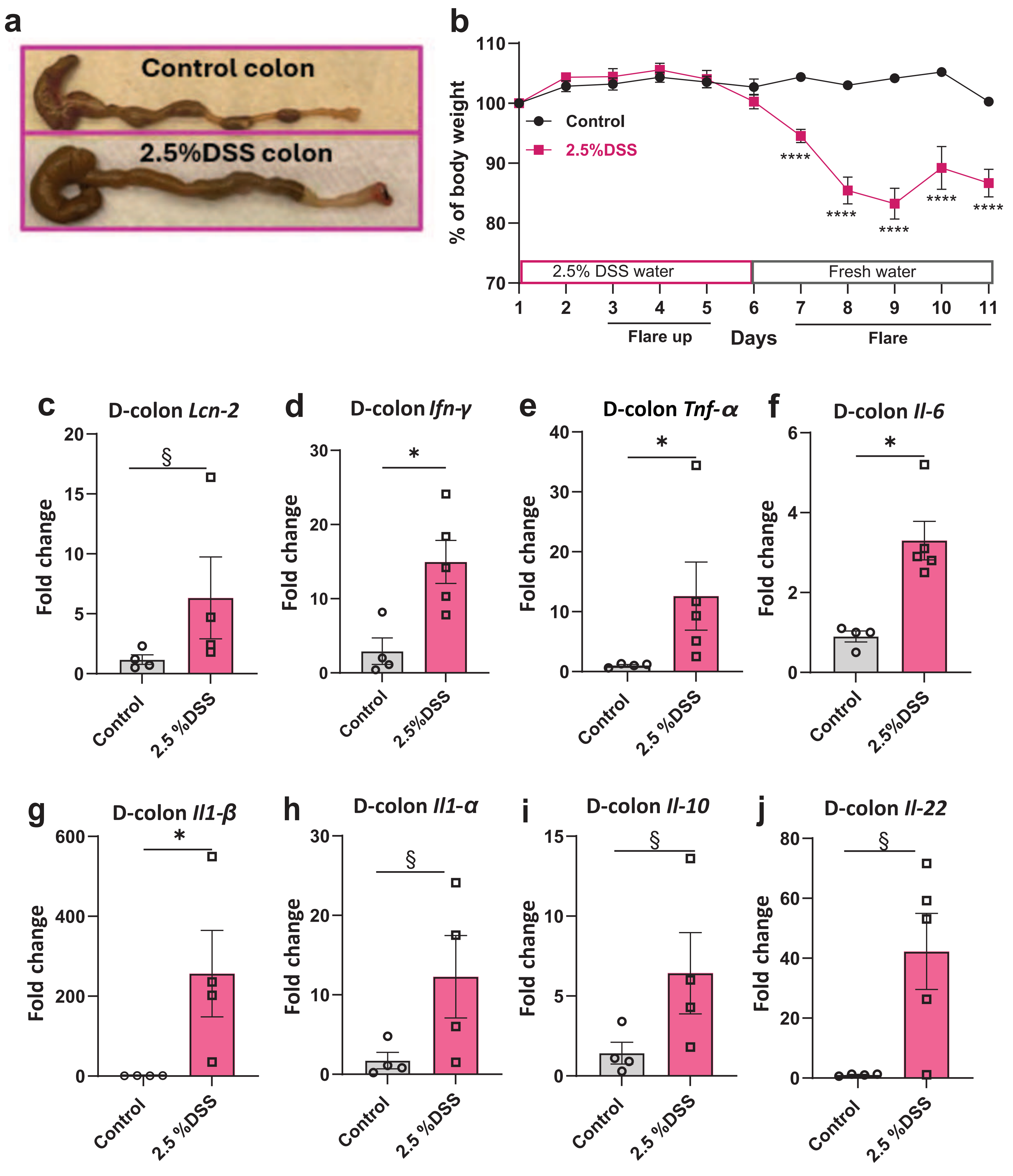

a
Control colon
2.5%DSS colon
b
% of body weight
Control
2.5%DSS
2.5% DSS water
Fresh water
Flare up
Days
Flare
****
c
D-colon Lcn-2
d
D-colon Ifn-γ
e
D-colon Tnf-α
f
D-colon Il-6
g
D-colon Il1-β
h
D-colon Il1-α
i
D-colon Il-10
j
D-colon Il-22
Fold change
Control
2.5 %DSS
§
*

Fig.S1| Assessment of colitis induction in mice.

(a) Representative image of a non-inflamed colon from control (day 8; upper panel) compared to an inflamed colon from mice treated with 2.5% DSS dissolved in drinking water (day 8; lower panel). (**b**) Percent change in body weight compared to initial body weight during experimental colitis induction. Inflammatory markers mRNA expression was measured by qRT-PCR in the distal colon tissues from DSS-treated mice during the active flare phase (days 8 and 11) and control mice, normalized to (*Tbp*); **(c)** Lipocalin-2 (*Lcn-2*), (**d**) Interferon gamma *(Ifn-γ)* (**e**) *Tumor necrosis factor alpha (Tnfα)*, (**f**) Interleukin 6 *(Il-6)*, (**g**) Interleukin-1 beta *(Il-1β)*, (**h**) Interleukin-1 alpha (*Il-1α*), and anti-inflammatory cytokines, (**i**) Interleukin-10 (*Il-10*) and (**j**) Interleukin-22 (*Il-22*). Data are presented as mean ± SEM, in (**b**) (n=4-12) and (**c-j**) (n=4-5). Mann-Whitney U test for two-groups comparisons. § <0.1, *P<0.05, **P<0.01,***P<0.001, and ****P<0.0001.

Fig.S2

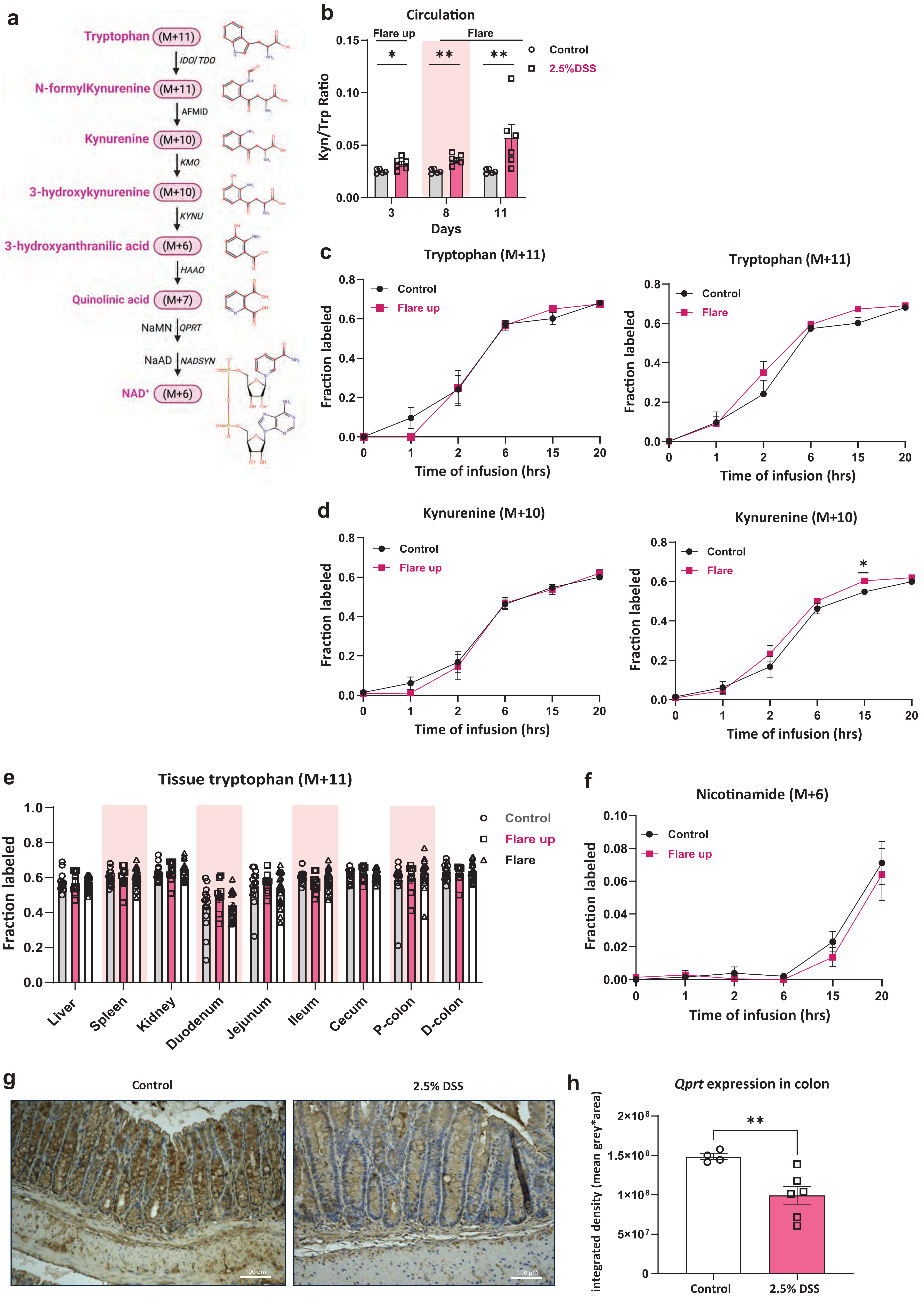
a
Tryptophan (M+11)
IDO/TDO
N-formylKynurenine (M+11)
AFMID
Kynurenine (M+10)
KMO
3-hydroxykynurenine (M+10)
KYNU
3-hydroxyanthranilic acid (M+6)
HAAO
Quinolinic acid (M+7)
NaMN QPRT
NaAD NADSYN
NAD+ (M+6)
b
Circulation
Flare up
Flare
Control
2.5%DSS
Kyn/Trp Ratio
Days
c
Tryptophan (M+11)
Fraction labeled
Control
Flare up
Flare
Time of infusion (hrs)
d
Kynurenine (M+10)
e
Tissue tryptophan (M+11)
Liver
Spleen
Kidney
Duodenum
Jejunum
Ileum
Cecum
P-colon
D-colon
f
Nicotinamide (M+6)
g
Control
2.5% DSS
100 μm
h
Qprt expression in colon
integrated density (mean grey*area)
Control
2.5% DSS

Fig.S2| Metabolic analysis of tryptophan and its downstream metabolites in the serum and tissues during acute intestinal inflammation.

(a) Schematic of tryptophan metabolism through the kynurenine pathway (M+n) M represents the mass of the labeled metabolite and the n is the number of labeled carbons. (**b**) LC-MS measurement of the total abundance of the kynurenine-to-tryptophan ratio (Kyn/Trp) in the circulation of mice treated with 2.5% DSS compared to control mice (n=4-5). (**c-d**) LC-MS analysis of labeled fractions of tryptophan and kynurenine in the serum during the early flare up (left) and active flare (right) phases. (**e**) Tissue fraction labeled of tryptophan. (**f**) Serum fractional labeling of nicotinamide over 20-hour intravenous infusion during the early flare up phase of intestinal inflammation. (**g**) Immunohistochemical (IHC) staining of *Qprt* in Swiss roll sections of the distal colon from 2.5% DSS-treated mice and control (at magnification 40x, scale bar 100μm). (**h**) Quantification of *qprt* expression in 2.5% DSS-treated and control groups. Data are presented as mean ± SEM; in (**a**) (n=6 in each group), (**c-f**) (n=10-20). Statistical significance was determined by Mann-Whitney U test used for comparisons between two groups, and the Kruskal-Wallis test followed by Dunn's post hoc test for comparisons among more than two groups. $^*P<0.05$ and $^{**}P<0.01$.

Fig.S3

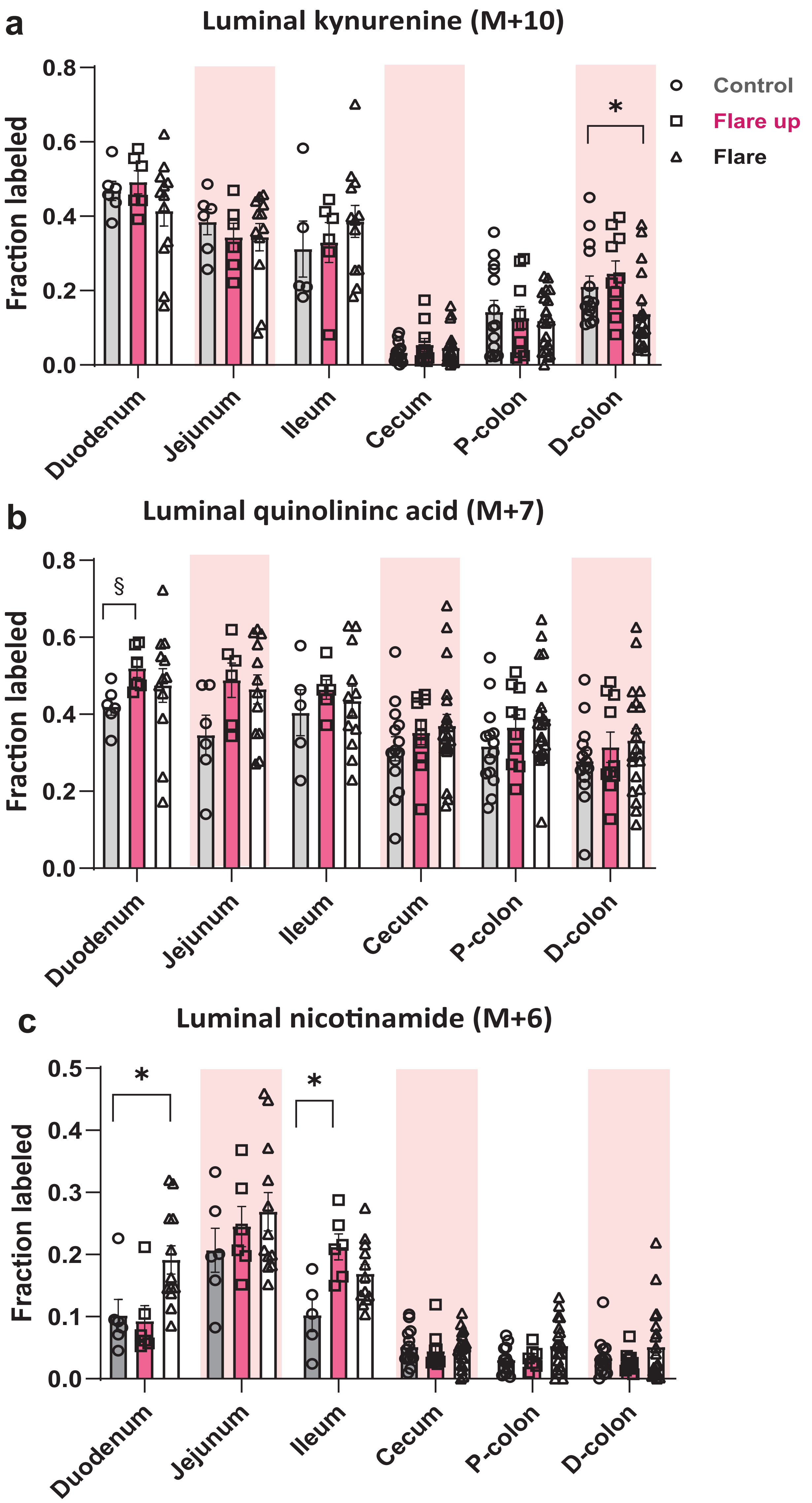
a
Luminal kynurenine (M+10)
Fraction labeled
0.0
0.2
0.4
0.6
0.8
Control
Flare up
Flare
*
Duodenum
Jejunum
Ileum
Cecum
P-colon
D-colon
b
Luminal quinolininc acid (M+7)
Fraction labeled
0.0
0.2
0.4
0.6
0.8
§
Duodenum
Jejunum
Ileum
Cecum
P-colon
D-colon
c
Luminal nicotinamide (M+6)
Fraction labeled
0.0
0.1
0.2
0.3
0.4
0.5
*
*
Duodenum
Jejunum
Ileum
Cecum
P-colon
D-colon

Fig.S3| Gut luminal tryptophan downstream metabolites via the kynurenine pathway.

The fractional labeled of luminal metabolites derived from tryptophan tracer measured by LC-MS of (**a**) kynurenine, (**b**) quinolinic acid, (**c**) nicotinamide in DSS-treated mice compared to control. Data are presented as mean ± SEM (n=10-20). Statistical significance was determined by Kruskal-Wallis test followed by Dunn's post hoc test for comparisons among more than two groups. § <0.1 and *P<0.05.

Fig.S4

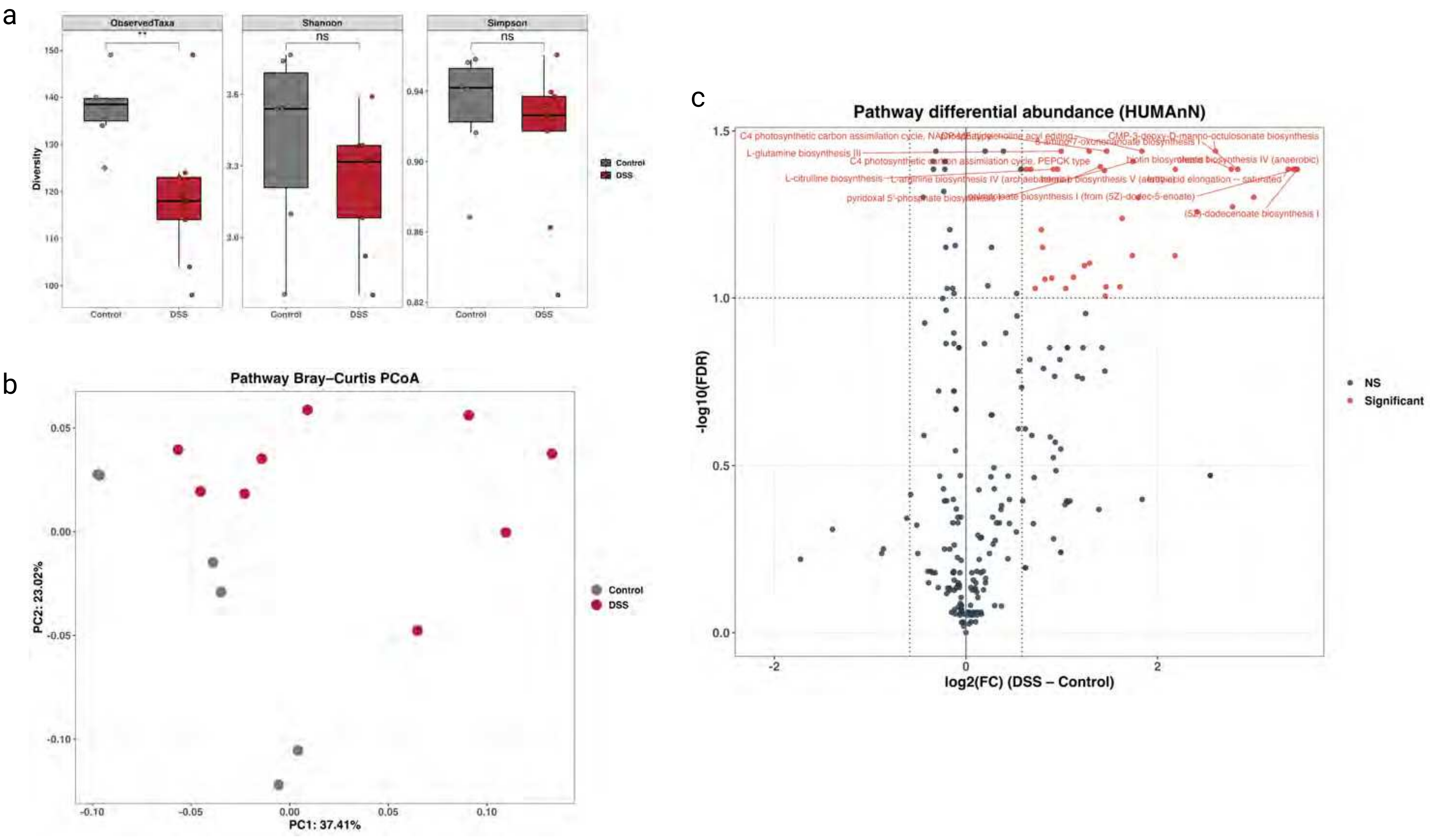

a
ObservedTaxa
Shannon
Simpson
Diversity
Control
DSS
**
ns
ns
b
Pathway Bray–Curtis PCoA
PC1: 37.41%
PC2: 23.02%
Control
DSS
c
Pathway differential abundance (HUMAnN)
L-glutamine biosynthesis III
L-citrulline biosynthesis
CMP-3-deoxy-D-manno-octulosonate biosynthesis
(5Z)-dodecenoate biosynthesis I
log2(FC) (DSS – Control)
-log10(FDR)
NS
Significant

Fig.S4| Pathway-level functional diversity and differential abundance analysis of DSS-induced colitis microbiomes.

(a) Alpha-diversity indices calculated using species-level taxonomic abundances (Observed Species Richness, Shannon's, Simpson's) show a significant reduction in observed richness in DSS-treated mice during the active phase (days 8 and 11) ($p = 0.0097$, Welch's t-test) but no significant change observed in Shannon or Simpson diversity, indicating loss of low-abundance species without major shifts in evenness. **(b)** Principal coordinate analysis (PCoA) of Bray-Curtis dissimilarities reveals clear separation between control (gray) and DSS (red) groups (PERMANOVA, $R^2 = 0.21$, $p = 0.007$), suggesting significant restructuring of pathway-level functional potential. **(c)** Volcano plot of differential pathway abundance (HUMAnN) comparing DSS versus control groups. Of all pathways analyzed, significantly altered features ($FDR < 0.1$, $|log_2FC| > 0.5$) are highlighted in red.

Fig.S5

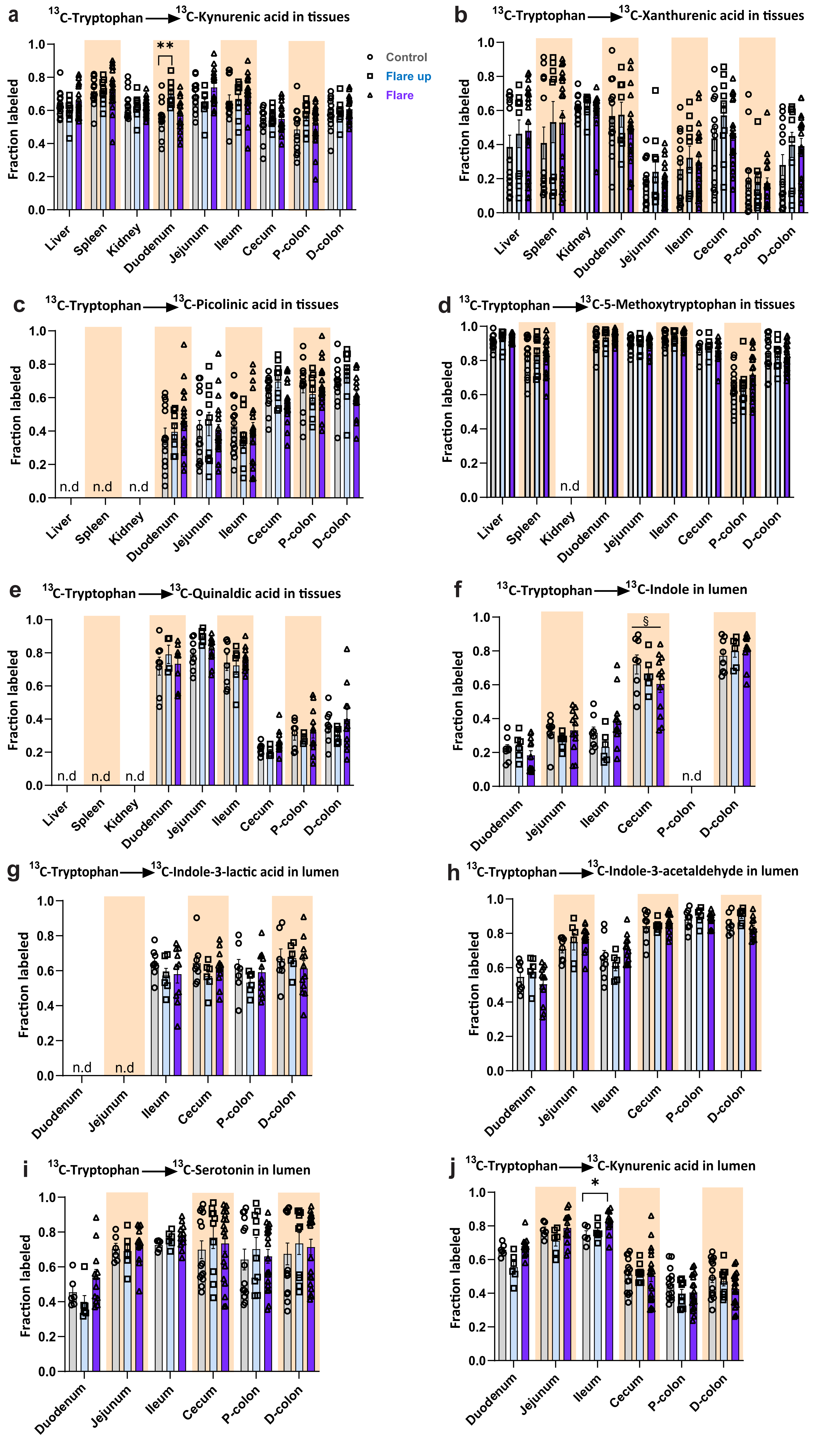
a
13C-Tryptophan → 13C-Kynurenic acid in tissues
Control
Flare up
Flare
**
Fraction labeled
1.0
0.8
0.6
0.4
0.2
0.0
Liver
Spleen
Kidney
Duodenum
Jejunum
Ileum
Cecum
P-colon
D-colon
b
13C-Tryptophan → 13C-Xanthurenic acid in tissues
c
13C-Tryptophan → 13C-Picolinic acid in tissues
n.d
n.d
n.d
d
13C-Tryptophan → 13C-5-Methoxytryptophan in tissues
n.d
e
13C-Tryptophan → 13C-Quinaldic acid in tissues
n.d
n.d
n.d
f
13C-Tryptophan → 13C-Indole in lumen
§
n.d
g
13C-Tryptophan → 13C-Indole-3-lactic acid in lumen
n.d
n.d
h
13C-Tryptophan → 13C-Indole-3-acetaldehyde in lumen
i
13C-Tryptophan → 13C-Serotonin in lumen
j
13C-Tryptophan → 13C-Kynurenic acid in lumen
*

Fig.S5| Quantification of tryptophan-derived pathways in host tissues and gut lumen.

Tracing the incorporation of ($^{13}C_{11}$-tryptophan) into different tryptophan-derived metabolites following 20-hour intravenous infusion, measured by LC-MS in the host tissues, (**a**) kynurenic acid, (**b**) xanthurenic acid, (**c**) picolinic acid, (**d**) 5-methoxytryptophan, (**e**) quinaldic acid; and in the gut lumen, (**f**) indole, (**g**) indole-3-lactic acid, (**h**) indole-3-acetaldehyde, (**i**) serotonin, (**j**) kynurenic acid. Data are presented as mean ± SEM (n=10-20). Statistical significance was determined by Kruskal-Wallis test followed by Dunn's post hoc test for comparisons among more than two groups. § <0.1, *P<0.05, **P<0.01, ***P<0.001, and ****P<0.0001. nd= not detected.

Fig.S6

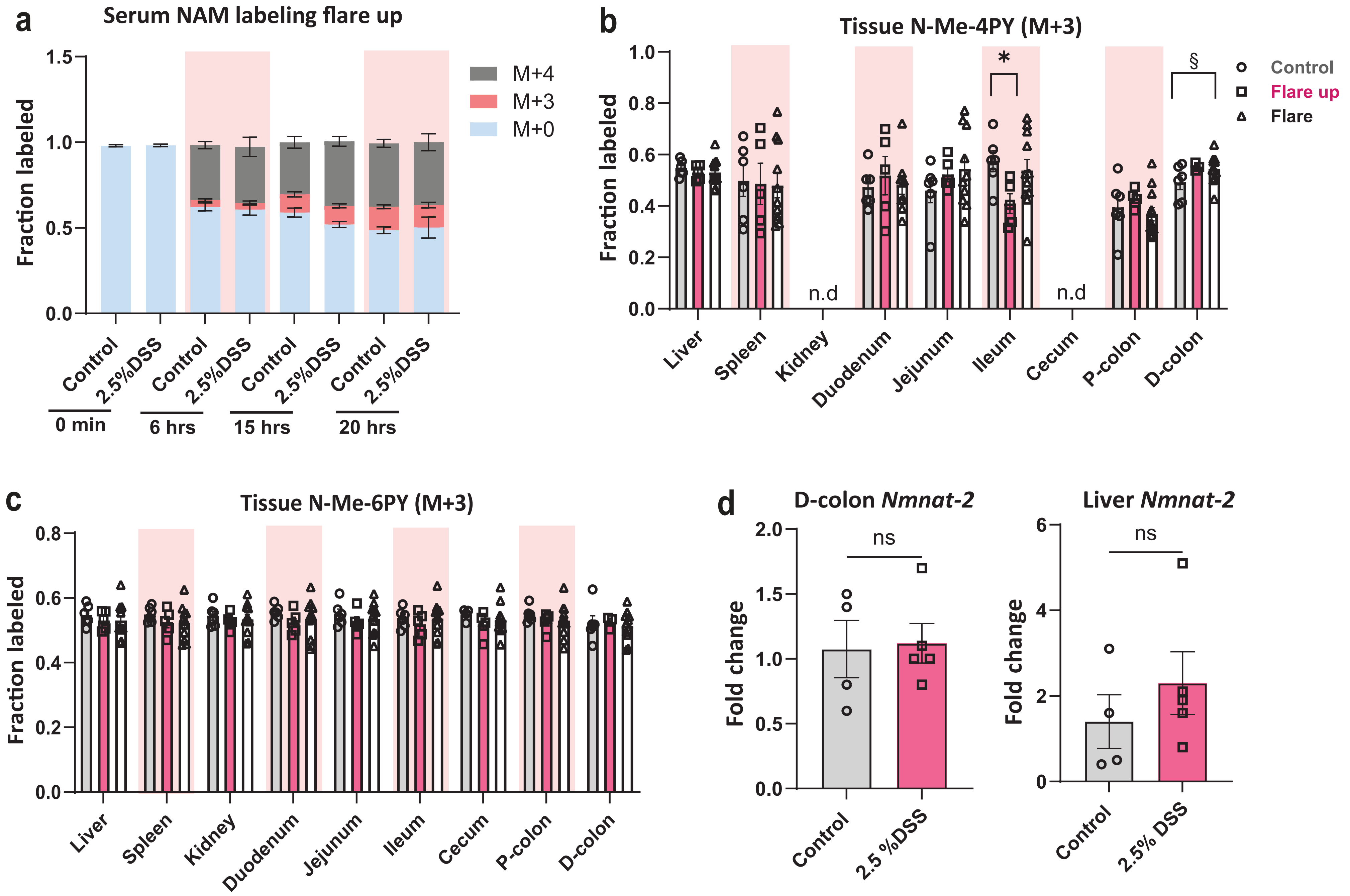
a
Serum NAM labeling flare up
M+4
M+3
M+0
Fraction labeled
1.5
1.0
0.5
0.0
Control
2.5%DSS
Control
2.5%DSS
Control
2.5%DSS
Control
2.5%DSS
0 min
6 hrs
15 hrs
20 hrs
b
Tissue N-Me-4PY (M+3)
Fraction labeled
1.0
0.8
0.6
0.4
0.2
0.0
*
§
n.d
n.d
Control
Flare up
Flare
Liver
Spleen
Kidney
Duodenum
Jejunum
Ileum
Cecum
P-colon
D-colon
c
Tissue N-Me-6PY (M+3)
Fraction labeled
0.8
0.6
0.4
0.2
0.0
Liver
Spleen
Kidney
Duodenum
Jejunum
Ileum
Cecum
P-colon
D-colon
d
D-colon Nmnat-2
Fold change
2.0
1.5
1.0
0.5
0.0
ns
Control
2.5 %DSS
Liver Nmnat-2
Fold change
6
4
2
0
ns
Control
2.5% DSS

Fig.S6| Metabolic profiling of metabolites produced via the salvage pathway from nicotinamide.

(a) Fractional labeling of nicotinamide in the circulation over 20-hour intravenous infusion of (2,4,5,6-$^2H_4$-NAM) during the early flare up phase (M+0: unlabeled NAM, M+3: recycled NAM, M+4: infusate NAM). Fraction labeled of the methylnicotinamide byproducts in host tissues, (**b**) N-Me-4PY and (**c**) N-Me-6PY. (**d**) qRT-PCR analysis of mRNA expression normalized to *Tbp* in distal colon and liver tissues during the active flare phase (days 8 and 11) compared to control of nicotinamide mononucleotide adenylyltransferase 2 (*NMNAT 2*; cytoplasmic). Data are presented as mean ± SEM, in **a-c** (n=8-18) and in **d** (n=45). Statistical significance was determined by Mann-Whitney U test used for comparisons between two groups, and the Kruskal-Wallis test followed by Dunn's post hoc test for comparisons among more than two groups. § <0.1, *P<0.05, ns= not significant.

Fig.S7

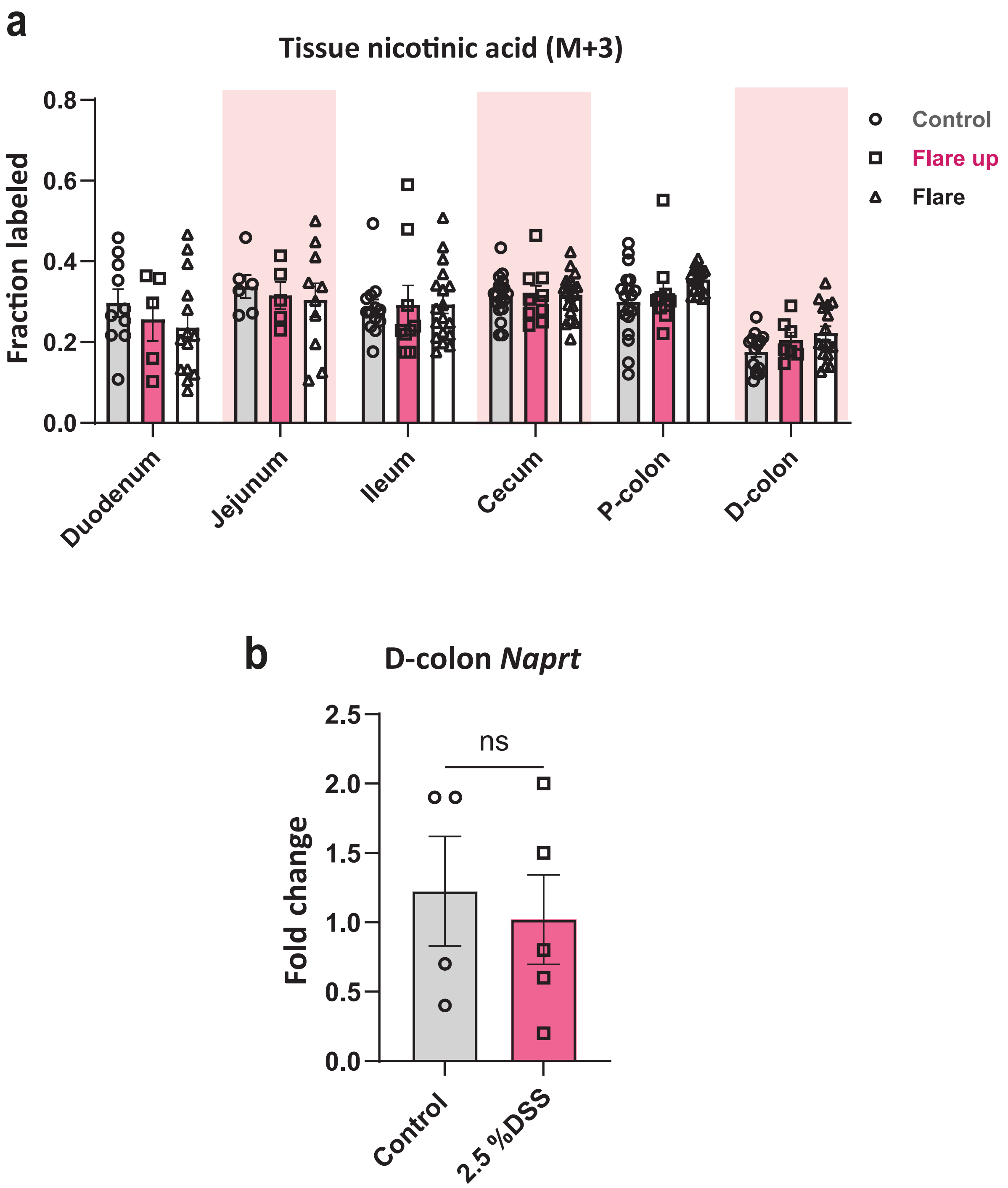
a
Tissue nicotinic acid (M+3)
Fraction labeled
0.8
0.6
0.4
0.2
0.0
Duodenum
Jejunum
Ileum
Cecum
P-colon
D-colon
Control
Flare up
Flare
b
D-colon *Naprt*
Fold change
2.5
2.0
1.5
1.0
0.5
0.0
ns
Control
2.5 %DSS

Fig.S7| Flux from microbial nicotinic acid to host tissues.

(a) Tissues fractional labeled of nicotinic acid produced from microbial nicotinic acid (n=8-18). (**b**) qRT-PCR analysis of mRNA expression normalized to *Tbp* in the distal colon of nicotinate phosphoribosyltransferase (*Naprt*) during the active phase of DSS-induced colitis (days 8 and 11) compared to control (n=4-5). Data are presented as mean ± SEM. Statistical significance was determined by Mann-Whitney U test used for comparisons between two groups, and the Kruskal-Wallis test followed by Dunn's post hoc test for comparisons among more than two groups. ns= not significant.

Fig.S8

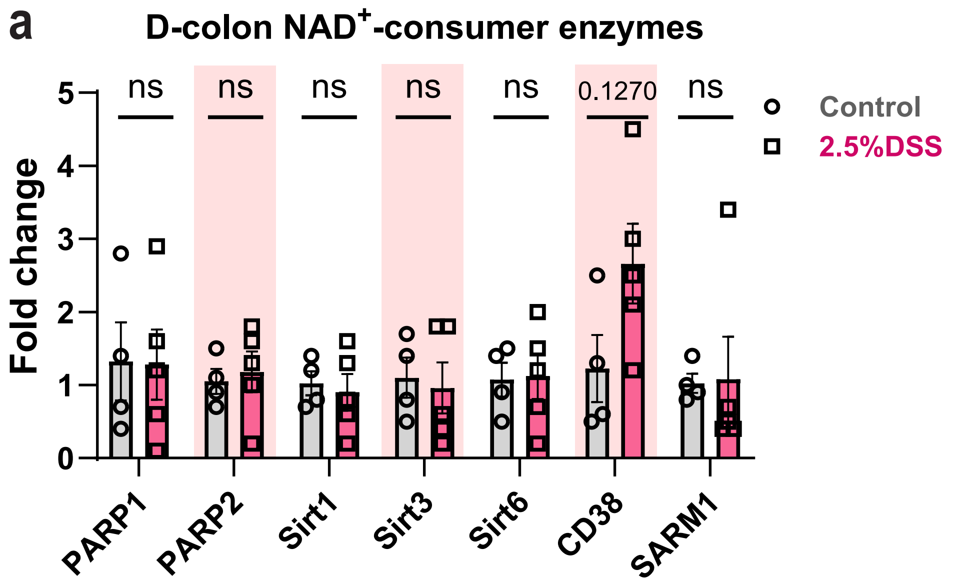
a
D-colon NAD+-consumer enzymes
Fold change
0
1
2
3
4
5
ns
ns
ns
ns
ns
0.1270
ns
PARP1
PARP2
Sirt1
Sirt3
Sirt6
CD38
SARM1
Control
2.5%DSS

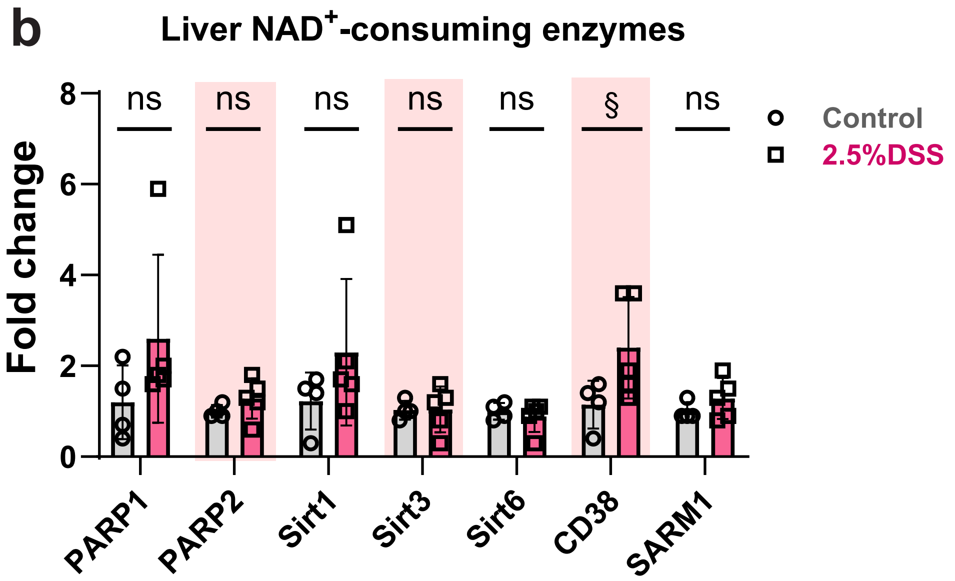
b
Liver NAD+-consuming enzymes
Fold change
0
2
4
6
8
ns
ns
ns
ns
ns
§
ns
PARP1
PARP2
Sirt1
Sirt3
Sirt6
CD38
SARM1
Control
2.5%DSS

Fig.S8| Analysis of the activity of NAD$^+$-consuming enzymes during intestinal inflammation.

Analysis of mRNA expression measured by qRT-PCR and normalized to *Tbp* in tissues (**a**) distal colon and (**b**) liver; left to right, poly ADP-ribose polymerase (*PARP 1-2*), sirtuins (*Sirt 1,3,6*), *CD38,* sterile alpha and toll/interleukin-1 receptor motif-containing 1 (*SARM1*) during the active phase of DSS-induced colitis (days 8 and 11) compared to control. Data are presented as mean ± SEM, (n=4-5). Statistical significance was determined by Mann-Whitney U test used for comparisons between two groups. § <0.1, ns= not significant.

Supplemental table 1: Primers used for gene expression analysis

Target gene	Species	Taqman ID	RefSeq number
Ido1	Mouse	Mm00492590_m1	NM_001293690.1
Ido2	Mouse	Mm00524210_m1	NM_145949.2
Tdo2	Mouse	Mm01220281_m1	NM_019911.2
Nampt	Mouse	Mm00451938_m1	NM_021524.2
Nmnat1	Mouse	Mm01257929_m1	NM_133435.1
Nmnat2	Mouse	Mm00615393_m1	NM_175460.3
Nmnat3	Mouse	Mm00513791_m1	NM_144533.2
Nnmt	Mouse	Mm00447994_m1	NM_010924.2
Naprt	Mouse	Mm00553802_m1	NM_172607.3
Parp1	Mouse	Mm01321084_m1	NM_007415.2
Parp2	Mouse	Mm01319555_m1	NM_009632.2
Sirt1	Mouse	Mm01168521_m1	NM_001159589.1
Sirt3	Mouse	Mm00452131_m1	NM_001127351.1
Sirt6	Mouse	Mm01149042_m1	NM_001163430.1
Sarm1	Mouse	Mm00555617_m1	NM_001168521.1
CD38	Mouse	Mm01220904_m1	NM_007646.4
Il-6	Mouse	Mm00446190_m1	NM_031168.1
Il-1α	Mouse	Mm00439620_m1	NM_010554.4
Il-1β	Mouse	Mm00434228_m1	NM_008361.3
Tnf-α	Mouse	Mm00443258_m1	NM_001278601.1
Ifn-γ	Mouse	Mm01168134_m1	NM_008337.3
Il-10	Mouse	Mm00439614_m1	NM_010548.2
Il-22	Mouse	Mm01226722_g1	NM_016971.2
Lcn-2	Mouse	Mm01324470_m1	NM_008491.1
Ocln	Mouse	Mm00500910_m1	NM_008756.2
Tbp	Mouse	Mm00446971_m1	NM_013684.3
